# Research on the dissemination mechanism of online rumors in public health events based on the SNITR model

**DOI:** 10.3389/fpubh.2026.1791115

**Published:** 2026-04-20

**Authors:** Mengna Zhang, Xin Zhang, Yuqing Chen, Fuzhao Li

**Affiliations:** 1 School of Management Science and Engineering, Guizhou University of Finance and Economics, Guiyang, China; 2 College of Public Administration, Guizhou University of Finance and Economics, Guiyang, China; 3 Party and Government Office, Guizhou University of Finance and Economics, Guiyang, China

**Keywords:** public crisis events, transmitter nodes, rumor propagation model, rumor-transmitter mechanism, equilibrium points

## Abstract

During public health events, nodes on social networks that are aware of the truth may still actively disseminate rumors, thereby influencing the rumor propagation process. However, existing research has not yet taken into account the impact of transmitter nodes on rumor dissemination. Therefore, our study proposes an SNITR rumor propagation model that incorporates a rumor-transmitter mechanism. When rumor information emerges on social networks, susceptible users may neglect to propagate the received rumor information upon exposure (neglected nodes), some users may believe the rumors and spread them (infected nodes), while others may publicly state the truth to reduce the spread of rumors (transmitter nodes). Eventually, users will enter a recovered state and no longer participate in the rumor propagation process. By introducing the rumor-transmitter mechanism, a competitive coupling mechanism among different user groups is established. Additionally, the dynamics of the rumor propagation model are analyzed, the system's equilibrium points and basic reproduction number are calculated, and it is demonstrated that the equilibrium points are both locally and globally asymptotically stable. Finally, simulation experiments validate the effectiveness of the theoretical model and demonstrate that it aligns well with the intrinsic mechanisms of actual rumor propagation.

## 1 Introduction

During public health events, the different states of users involved in rumor propagation significantly influence the forwarding behavior of rumors. Nekovee et al. (1) extended the classic Susceptible-Infected-Recovered (SIR) rumor propagation model by innovatively introducing the key factor of the forgetting mechanism. They theoretically derived the dynamic laws of this model and obtained the patterns of rumor information dissemination through numerical simulations. However, they did not consider memory factors. Mortaji et al. (2) constructed a rumor propagation model that integrates “skepticism mechanisms” and “immunization mechanisms,” systematically incorporating multidimensional influencing factors within the framework of group behavior evolution. Yan et al. (3) analyzed the characteristics of rumor dissemination on social media, innovatively drew on herd immunity theory from infectious disease dynamics, and introduced a network self-purification mechanism into rumor propagation modeling. Through rigorous mathematical derivation and simulation validation, they successfully solved for the critical threshold of rumor propagation. Zhou et al. (4) proposed a resonance mechanism and compared the differences in dissemination mechanisms. Building on this, they introduced the SCIR (Susceptible-Chord-Infected-Recovered) rumor propagation model and, by incorporating social psychology dimensions, innovatively discovered the regulatory role of empathy in rumor dissemination. Pacheco, Ian Sa et al. (5) proposed the NI-SIR (Networked Infected-Susceptible-Infected- Recovered) model, which broke through traditional assumptions by incorporating node influence disparities for the first time. By establishing a node influence grading system, they achieved dynamic regulation of contact infection and recovery probabilities, making the model more aligned with the heterogeneous characteristics of real social networks.

The aforementioned studies investigate rumor propagation patterns based on different network structures and user states. However, in the era of information overload, each user develops their own thoughts after receiving rumors, which may include nodes that neglect to believe the rumor and nodes that identify the rumor and still transmit it. Current research has not considered the impact of truth spreaders (transmitter nodes) and neglected individuals (neglected nodes) on rumor propagation nor have they integrated infected nodes and transmitter spreaders to predict the trend of rumor dissemination. Building on the above studies, the study introduces neglected nodes and transmitter nodes and constructs a new rumor propagation model by combining infected nodes and transmitter nodes, aiming to more accurately describe the patterns of rumor dissemination in social networks.

Social media allows users to express viewpoints (including opposing viewpoints), so there are users who transmit rumors and various forms of user existence. Ghosh et al. (6) considered the counterattack mechanism in rumor propagation, categorizing social media users into four types, namely susceptible nodes, infected nodes, counter-rumor spreading nodes, and recovered nodes, and studied both deterministic and stochastic models. They explored the conditions for rumor neglect and extinction through numerical simulations and sensitivity analysis. Zan et al. (7) proposed the SICR (Susceptible-Infected-Carrier-Recovered) model based on the SIR model. They studied the peak and final scale of rumor propagation with parameters and constructed a rumor propagation model related to counterattack intensity. Xiao et al. (8) considered the competition between rumors and counter-rumor information, combining user behavior and external factors to construct the SKIR (Susceptible- Knowledgeable-Infected-Recovered) model based on evolutionary game theory and the SIR model.

The aforementioned studies are significant for exploring the patterns of rumor information dissemination but do not consider the influence of truth spreaders on rumor propagation. Zhang et al. (9) introduced truth spreaders into the rumor propagation process, establishing the SITR (Susceptible-Infected-Truth-Recovered) model. They proved the existence and stability conditions of boundary equilibrium points and validated the theoretical results through numerical simulations. Lin et al. (10) established an SWIR (Susceptible-Wavering-Infected-Recovered) model for disinformation diffusion, introducing malicious spreaders to study the diffusion trends of disinformation and the stability of the dynamic model. The above studies discuss the influence of truth spreaders or malicious spreaders on rumor propagation but do not combine these two types of spreaders in their research, and they rarely predict the trends of rumor propagation.

To address the shortcomings of the above research, the study establishes a new rumor propagation model. This model introduces neglected and transmitter nodes. Considering the guiding role of transmitter nodes on other nodes in different states, our study refers to this as the counter-rumor mechanism. The equilibrium points of the model are calculated, and the local asymptotic stability of these equilibrium points is proven. In addition to the traditional rumor-free equilibrium and endemic equilibrium, there are two boundary equilibria that reflect the competitive state between the counter-rumor mechanism and rumor propagation, making the rumor propagation scenarios more aligned with reality.

## 2 Method

### 2.1 Equations of the SNITR

Domestic and foreign scholars widely adopt epidemiological models to study the mechanisms of online rumor propagation, including the SIS (Susceptible-Infected-Susceptible) model, the SIR model, and the SEIR (Susceptible-Exposed-Infected-Recovered) model. According to the life cycle theory of information on social networks, information must undergo life processes such as gestation, generation, dissemination, and demise (11, 12), and the information dissemination process is time-sensitive. The SEIR model provides a more detailed division of the propagation stages, which helps to gain a deeper understanding of the mechanisms behind rumor spread. Therefore, the information propagation process in social networks is explained based on the SEIR model. In the SEIR model, nodes are categorized into four states: susceptible nodes (S), exposed nodes (E), infected nodes (I), and recovered nodes (R).

**Figure 1:**
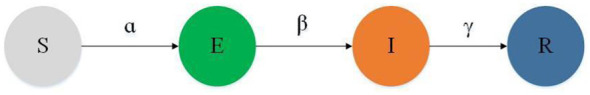
The state transition diagram of the SEIR mode.

The study adopts a continuous-time modeling framework and describes the evolution of the density of nodes in each state based on differential equations. In the adopted SEIR model, all transition parameters are rate parameters (transition proportion per unit time). When an infected node encounters a susceptible node, the proportion of susceptible nodes that transition to the exposed state is α, and the proportion of exposed nodes that transition to the infected state is β, meanwhile, the proportion of infected nodes that are cured and become recovered nodes is γ. The state transition diagram of the SEIR model is shown in Figure 1.

**Figure 2:**
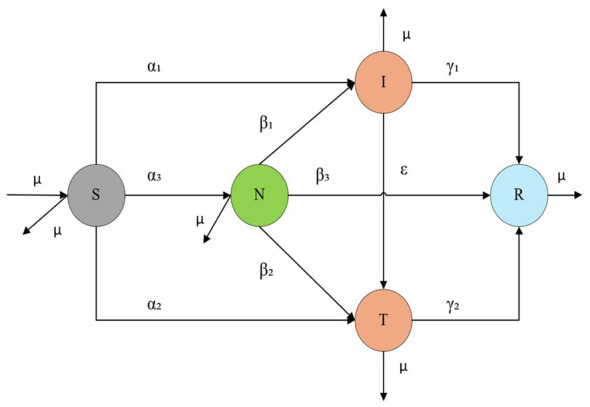
The SNITR model for rumor propagation.

In the dynamic process of rumor propagation, some nodes are able to identify the truth and still actively transmit rumors. Existing propagation models have not accounted for the dynamic impact of transmitter nodes on rumor spread. Therefore, the study introduces transmitter nodes and constructs a new rumor information propagation model—the SNITR model, as illustrated in Figure 2.

The SNITR model categorizes users into five types:

(1) Susceptible nodes (S) represent nodes that are unaware of the rumor. (2) Neglected nodes (N) represent nodes that neglect to spread the rumor. (3) Infected nodes (I) represent nodes that believe and actively spread the rumor. (4) Transmitter nodes (T) represent nodes that dare to publicly clarify the truth. (5) Recovered nodes (R) represent nodes that have ceased spreading the rumor.

Real social networks are dynamic: users may join or leave at any time, leading to continuous evolution of the network structure. To enhance the practical applicability of the model, the study constructs an open social network propagation framework based on the SNITR model. It is assumed that users register and log out at a constant rate μ.

During the propagation of rumor information, some infected nodes (I) believe and spread the rumor. However, influenced by truthful information, they may begin to doubt the authenticity of the rumor, leading to an uncertain attitude toward it, thus transitioning into neglected nodes (N). Meanwhile, some transmitter nodes (T), which dare to publicly clarify the truth and reduce the spread of rumors, may be repeatedly exposed to rumor information. As a result, a portion of these nodes may develop an uncertain attitude toward the rumor and transition into neglected nodes (N), while another portion may come to believe the rumor and start spreading it, transitioning into infected nodes (I). Based on the above rumor propagation process, the transition rules between different user states in the SNITR model are defined as follows:

(1) When susceptible nodes (S) come into contact with rumor-spreading nodes, some nodes may believe and forward the rumor, and the proportion of nodes transitioning to the infected nodes (I) is α*1*. Other nodes may identify the rumor as false and dare to speak the truth to reduce its spread, and the proportion of nodes transitioning to the transmitter nodes (T) is α_2_. Additionally, some nodes may adopt an uncertain attitude toward the rumor, and the proportion of nodes transitioning to the neglected nodes (N) is α3, where α_1_, α_2_, α_3_∈(0, 1) and α_1_+α_2_+α_3_ ≤ 1.

(2) As the rumor spreads further, some neglected nodes(N), upon discovering that the rumor has been repeatedly forwarded, subjectively perceive it as true, believe it, and forward it; the proportion of nodes transitioning to the infected nodes (I) is β_1_. As the truth of the public health event emerges, some neglected nodes (N) identify the rumor; the proportion of nodes transitioning to the transmitter nodes (T) is β_2_. Some neglected nodes(N), influenced by forgetting factors, lose interest in the rumor and become unwilling to spread it; the proportion of nodes transitioning to the recovered nodes (R) is β_3_, where β_1_, β_2_, β_3_∈(0, 1) and β_1_+β_2_+β_3_ ≤ 1.

(3) Similar to the state transition of neglected nodes (N), infected nodes(I), influenced by forgetting factors, lose interest in the rumor and become unwilling to spread it, and the proportion of nodes transitioning to the recovered nodes (R) is γ_1_. Some infected nodes (I), due to changes in perception, identify the rumor and are willing to speak the truth, and the proportion of nodes transitioning to the transmitter nodes (T) is ε. Where γ_1_, ε∈(0, 1) and γ_1_+ε ≤ 1.

(4) The transmitter nodes (T) were influenced by forgetting factors, lose interest in the rumor, and become unwilling to spread it; the proportion of nodes transitioning to the recovered nodes (R) is γ_2_. where γ_2_∈(0, 1).

Based on the above transition rules among users in different states, a social network rumor propagation model is established as shown in Equation 1. The meanings of all parameters in the model are listed in Table 1.

**Table 1:** Meanings of parameters.

Parameter	Meaning
α_1_	The proportion of susceptible nodes that receive rumor information and convert into infected nodes.
α_2_	The proportion of susceptible nodes that receive rumor information and convert into transmitter nodes.
α_3_	The proportion of susceptible nodes that receive rumor information and convert into neglected nodes.
β_1_	The proportion of neglected nodes that receive rumor information and convert into infected nodes.
β_2_	The proportion of neglected nodes that receive rumor information and convert into transmitter nodes.
β_3_	The proportion of neglected nodes that receive rumor information and convert into recovered nodes.
γ_1_	The proportion of infected nodes that receive rumor information and convert into recovered nodes.
γ_2_	The proportion of transmitter nodes that receive rumor information and convert into recovered nodes.
ε	The proportion of infected nodes that receive rumor information and convert into transmitter nodes.

The dynamic differential equations of the improved SNITR model are shown in Equation 1. Assuming that the number of Weibo users changes dynamically over time, then at any time, S(t) + N(t) + I(t) + T(t) + R(t) = N(t).


$$\{\begin{array}{l} \frac{dS(t)}{dt}=\mu -\alpha_{1}I(t)S(t)-\alpha_{2}T(t)S(t)-\alpha_{3}S(t)N(t)-\mu S(t)\\ \frac{dN(t)}{dt}=\alpha_{3}N(t)S(t)-\beta_{1}I(t)N(t)-\beta_{2}T(t)N(t)-\beta_{3}N(t)\\ -\mu N(t)\\ \frac{dI(t)}{dt}=\alpha_{1}S(t)I(t)+\beta_{1}N(t)I(t)-\varepsilon I(t)-\gamma_{1}I(t)-\mu I(t)\\ \frac{dT(t)}{dt}=\alpha_{2}S(t)T(t)+\beta_{2}N(t)T(t)+\varepsilon I(t)-\gamma_{2}T(t)-\mu T(t)\\ \frac{dR(t)}{dt}=\gamma_{1}I(t)+\gamma_{2}T(t)+\beta_{3}N(t)-\mu R(t) \end{array}$$ (1)

Since the first four equations in Equation 1 are independent of the fifth equation, the fifth equation can be omitted. Therefore, System (1) can be simplified to an equivalent subsystem. The expression of the simplified system is shown in Equation 2 as follows:


(2) $$\{\begin{array}{l} \frac{dS(t)}{dt}=\mu -\alpha_{1}I(t)S(t)-\alpha_{2}T(t)S(t)-\alpha_{3}S(t)N(t)-\mu S(t)\\ \frac{dN(t)}{dt}=\alpha_{3}N(t)S(t)-\beta_{1}I(t)N(t)-\beta_{2}T(t)N(t)-\beta_{3}N(t)\\ -\mu N(t)\\ \frac{dI(t)}{dt}=\alpha_{1}S(t)I(t)+\beta_{1}N(t)I(t)-\varepsilon I(t)-\gamma_{1}I(t)-\mu I(t)\\ \frac{dT(t)}{dt}=\alpha_{2}S(t)T(t)+\beta_{2}N(t)T(t)+\varepsilon I(t)-\gamma_{2}T(t)-\mu T(t) \end{array}$$

Based on the rumor propagation model designed in our study, the equilibrium points of the model are first calculated. By studying the stable states of the system under specific conditions, a clear understanding of the potential stable states the rumor propagation system may reach under different scenarios can be obtained. Secondly, the basic reproduction number of the model is calculated, which helps determine the potential number of individuals that rumor spreaders may affect. Finally, the stability of the equilibrium points is proven. It allows us to predict in advance whether rumor propagation tends to fade away, persist in a stable state, or undergo new changes, thereby providing strong support for forecasting the trend of rumor propagation.

### 2.2 Calculation of model equilibrium points

Assuming the right-hand side of the differential equations in Equation 2 is set to zero, the non-negative equilibrium points of system (2) under different conditions are calculated. This allows for the investigation of the stable states that the rumor propagation system may achieve and the analysis of the propagation trends of rumors.

(1) When there are no neglected nodes N, infected nodes I, or transmitter nodes T in Equation 2, all nodes on social network are unaware nodes. At this point, the equilibrium point of the system is *E*_0_.

Since *N*_0_ = 0, *I*_0_ = 0, *T*_0_ = 0, we have *S*_0_ = 1. Thus, the equilibrium point is *E*_0_= (*S*_0_, *N*_0_, *I*_0_, *T*_0_) =(1, 0, 0, 0). At this stage, rumors in the social network are in a completely unpropagated state.

(2) When there are no neglected nodes in Equation 2, *N*_1_ = 0, Equation 2 can be simplified to a new system expression as shown in Equation 3:


$$\{\begin{array}{l} \frac{dS(t)}{dt}=\mu -\alpha_{1}I(t)S(t)-\alpha_{2}T(t)S(t)-\mu S(t)\\ \frac{dI(t)}{dt}=\alpha_{1}S(t)I(t)-\varepsilon I(t)-\gamma_{1}I(t)-\mu I(t)\\ \frac{dT(t)}{dt}=\alpha_{2}S(t)T(t)+\varepsilon I(t)-\gamma_{2}T(t)-\mu T(t) \end{array}$$ (3)

Let the right-hand side of Equation 3 be zero to calculate the equilibrium points of system (3) as *E*_1_ = (*S*_1_, 0, 0, *T*_1_). Here, $S_{1}=\frac{\gamma_{2}+\mu }{\alpha_{2}},N_{1}=0,I_{1}=0,T_{1}=\frac{\mu }{\gamma_{2}+\mu }-\frac{\mu }{\alpha_{2}}$.

(3) When there are no transmitter nodes, *T*_2_= 0, Equation 2 can be simplified to a new system expression as shown in Equation 4


$$\{\begin{array}{l} \frac{dS(t)}{dt}=\mu -\alpha_{1}I(t)S(t)-\alpha_{3}S(t)N(t)-\mu S(t)\\ \frac{dI(t)}{dt}=\alpha_{3}N(t)S(t)-\beta_{1}I(t)N(t)-\beta_{3}N(t)-\mu N(t)\\ \frac{dT(t)}{dt}=\alpha_{1}S(t)I(t)+\beta_{1}N(t)I(t)-\varepsilon I(t)-\gamma_{1}I(t)-\mu I(t) \end{array}$$ (4)

Let the right-hand side of Equation 4 be zero to calculate the first equilibrium point of system (4). Here *E*_2_ = (*S*_2_, 0, *I*_2_, 0), where $S_{2}=\frac{\gamma_{2}+\mu }{\alpha_{2}}$, *N*_2_ = 0, *T*_2_ = 0, $I_{2}=\frac{\mu \left(\alpha_{2}-\gamma_{2}-\mu \right)}{\alpha_{2}\left(\gamma_{2}+\mu \right)}$.

Calculating the second equilibrium point of system (4). Here *E*_3_ = (*S*_3_, *N*_3_, 0, 0), where $S_{3}=\frac{\beta_{3}+\mu }{\alpha_{3}}$, $N_{3}=\frac{\mu \left(\alpha_{3}-\beta_{3}-\mu \right)}{\alpha_{3}\left(\beta_{3}+\mu \right)}$, *I*_3_ = 0, *T*_3_ = 0.

(4) When infected nodes and transmitter nodes exist in Equation 2, *N*_4_ = 0. The system (2) can be simplified into a new expression as shown in Equation 5:


$$\{\begin{array}{l} \frac{dS(t)}{dt}=\mu -\alpha_{1}I(t)S(t)-\alpha_{2}T(t)S(t)-\mu S(t)\\ \frac{dI(t)}{dt}=\alpha_{1}S(t)-\varepsilon -\gamma_{1}-\mu \\ \frac{dT(t)}{dt}=\alpha_{2}S(t)T(t)+\varepsilon I(t)-\gamma_{2}T(t)-\mu T(t) \end{array}$$ (5)

Calculating the equilibrium points of system (5) is *E*_4_ = (*S*_4_, 0, *I*_4_, *T*_4_). where $S_{4}=\frac{\varepsilon +\gamma_{1}+\mu }{\alpha_{1}}$, *N*_4_ = 0, $I_{4}=\mu \cdot \frac{1-S_{4}}{S_{4}}\cdot \frac{\gamma_{2}+\mu -\alpha_{2}S_{4}}{\alpha_{1}\left(\gamma_{2}+\mu -\alpha_{2}S_{4}\right)+\alpha_{2}\varepsilon }$,$T_{4}=\mu \cdot \frac{1-S_{4}}{S_{4}}\cdot \frac{\varepsilon }{\alpha_{1}\left(\gamma_{2}+\mu -\alpha_{2}S_{4}\right)+\alpha_{2}\varepsilon }$.

When neglected nodes and transmitter nodes exist in Equation 2, *I*_5_ = 0, Equation 2 can be simplified to a new system expression as shown in Equation 6:


$$\{\begin{array}{l} \frac{dS(t)}{dt}=\mu -\alpha_{2}T(t)S(t)-\alpha_{3}S(t)N(t)-\mu S(t)\\ \frac{dN(t)}{dt}=\alpha_{3}S(t)-\beta_{2}T(t)-\beta_{3}-\mu \\ \frac{dT(t)}{dt}=\alpha_{2}S(t)T(t)+\beta_{2}N(t)T(t)+\varepsilon I(t)-\gamma_{2}T(t)-\mu T(t) \end{array}$$ (6)

Calculating the equilibrium points of system (6) is *E*_5_ = (*S*_5_, *N*_5_, 0, *T*_5_). where $S_{5}=\frac{\mu \beta_{2}}{\alpha_{3}\gamma_{2}+\alpha_{3}\mu +\mu \beta_{2}-\alpha_{2}\beta_{3}-\alpha_{2}\mu }$, $N_{5}=\frac{\gamma_{2}+\mu -\alpha_{2}S_{5}}{\beta_{2}}$. $T_{5}=\frac{\alpha_{3}S_{5}-\beta_{3}-\mu }{\beta_{2}}$.

(6) When neglected nodes, infected nodes, and transmitter nodes exist in Equation 2, we calculate the equilibrium points of the system as *E*_6_ = (*S*_6_, *N*_6_*I*_6_, *T*_6_). Where $N_{5}=\frac{\varepsilon +\gamma_{1}+\mu -\alpha_{1}S_{6}}{\beta_{1}}$, $S_{6}=\frac{\varepsilon \left(\beta_{3}+\mu +\beta_{2}\right)+\beta_{1}\left(\gamma_{2}+\mu \right)-\beta_{2}\left(\varepsilon +\gamma_{1}+\mu \right)}{\beta_{1}\alpha_{2}-\beta_{2}\alpha_{1}+\varepsilon \alpha_{3}}$, $T_{6}=\frac{\varepsilon }{\beta_{1}\left(\gamma_{2}+\mu -\alpha_{2}S-\beta_{2}N\right)+\beta_{2}\varepsilon }\left(\alpha_{3}S-\beta_{3}-\mu \right)$, $I_{6}=\frac{\gamma_{2}+\mu -\alpha_{2}S-\beta_{2}N}{\varepsilon }T_{6}$.

### 2.3 Calculation of the basic reproduction number of the model

The basic reproduction number *R*_0_ is a key indicator for analyzing the threshold of infectious disease transmission, and its concept has been extended to the study of rumor propagation dynamics (13, 14). In our study, *R*_0_ represents the average number of unaware nodes that can be influenced by each infected node within a social network. It determines the critical condition for a rumor outbreak (*R*_0_ > 1) or its tendency to die out (*R*_0_ < 1). In our study, the next-generation matrix method (15) is used to calculate the value of *R*_0_, providing a theoretical threshold basis for control strategies.

Let *X* = (*N*(*t*), *I*(*t*), *T*(*t*))^*T*^, we can obtain $\frac{dX}{dt}=\text{F}-\nu$, where:


$$\begin{array}{l} \;F=\left[\begin{array}{c} \alpha_{1}SI\\ \alpha_{2}ST+\beta_{2}NT+\varepsilon I\\ \alpha_{3}NS \end{array}\right],\\ \nu =\left[\begin{array}{c} \left(\varepsilon +\gamma_{1}+\mu \right)I-\beta_{1}NI\\ \left(\gamma_{2}+\mu \right)T\\ \left(\beta_{1}I+\beta_{2}T\right)N+\left(\beta_{3}+\mu \right)N \end{array}\right]. \end{array}$$

Let ***F*** and ***v*** denote the Jacobian matrix of *F* and *v* at the rumor-free equilibrium point *E*_0_, respectively. Their calculations are as follows:


$$\begin{array}{l} F=\left[\begin{array}{ccc} \alpha_{1} & 0 & 0\\ 0 & \alpha_{2} & 0\\ 0 & 0 & \alpha_{3} \end{array}\right],\nu =\left[\begin{array}{ccc} \varepsilon +\gamma_{1}+\mu & 0 & 0\\ -\varepsilon & \gamma_{2}+\mu & 0\\ 0 & 0 & \beta_{3}+\mu \end{array}\right] \end{array}$$

Therefore, the next-generation matrix is given by *K* = **Fv**^−1^, and its calculation is shown in Equation 7:


$$\begin{array}{l} K=F\nu^{-1}=\left[\begin{array}{ccc} \frac{\alpha_{1}}{\varepsilon +\gamma_{1}+\mu } & 0 & 0\\ \frac{\alpha_{2}+\varepsilon }{\left(\varepsilon +\gamma_{1}+\mu \right)\left(\gamma_{2}+\mu \right)} & \frac{\alpha_{2}}{\gamma_{2}+\mu } & 0\\ 0 & 0 & \frac{\alpha_{3}}{\beta_{3}+\mu } \end{array}\right] \end{array}$$ (7)

The eigenvalues of *K* are its diagonal elements. Thus, the three basic reproduction numbers are given by Equations 8–10 respectively:


$$\begin{array}{l} R_{01}=\frac{\alpha_{1}}{\varepsilon +\gamma_{1}+\mu } \end{array}$$ (8)

$$\begin{array}{l} R_{02}=\frac{\alpha_{2}}{\gamma_{2}+\mu } \end{array}$$ (9)

$$\begin{array}{l} R_{03}=\frac{\alpha_{3}}{\beta_{3}+\mu } \end{array}$$ (10)

### 2.4 The equilibrium point is locally asymptotically stable

Based on the Lyapunov stability theorem (16) and the Routh-Hurwitz stability criterion (17), the local asymptotic stability of the seven equilibrium points of the SNITR model is proved below.

**(1) At the rumor-free equilibrium point** ***E***_0_,**the system (2) is locally asymptotically stable**.

**Theorem 1** If *R*_0_ < 1, then the system (2) is locally asymptotically stable at the rumor-free equilibrium point *E*_0_.

**Proof** Based on the next-generation matrix method (18, 19), the Jacobian matrix of system (2) at the rumor-free equilibrium point *E*_0_ can be obtained as shown in Equation 11:


$$\begin{array}{l} J_{\left(E_{0}\right)}=\left(\begin{array}{cccc} -\mu & 0 & -\alpha_{1} & -\alpha_{2}\\ 0 & \alpha_{3}-\beta_{3}-\mu & 0 & 0\\ 0 & 0 & \alpha_{1}-\varepsilon -\gamma_{1}-\mu & 0\\ 0 & 0 & \varepsilon & \alpha_{2}-\gamma_{2}-\mu \end{array}\right) \end{array}$$ (11)

The eigenvalues of the matrix *J*_(_*E*__0_)_ can be expressed as λ_1_ = −μ, λ_2_ = α_3_−β_3_−μ, λ_3_ = α_1_−ε−γ_1_−μ, and λ_4_ = α_2_−γ_2_−μ. Obviously λ_1_ = −μ < 0 holds. If *R*_0_ < 1, then *R*__0_1_ < 1, *R*__0_2_ < 1, and *R*__0_3_ < 1. Therefore, λ_2_ = α_3_−β_3_−μ < 0 hold. Thus, all three eigenvalues of the matrix *J*_(_*E*__0_)_ are negative real numbers. According to the Lyapunov stability theorem (16), the rumor-free equilibrium point *E*_0_ is locally asymptotically stable.

**(2) The system (2) is locally asymptotically stable at the infection-free equilibrium point** ***E***_1_

During rumor propagation, the user population may consist of infected nodes and transmitter nodes, with no infected nodes leading to a slower spread of rumor information. Under this scenario, the equilibrium points of the model are denoted as *E*_1_ and *E*_5_. Below is the proof of the stability of equilibrium point. Next, we prove the stability of the equilibrium point.

**Theorem 2** If *R*_01_ < 1 and *R*_02_ < 1, then system (2) is locally asymptotically stable at the rumor equilibrium point *E*_1_.

**Proof** The Jacobian matrix of system (2) at the rumor equilibrium point *E*_1_ is given by Equation 12:


$$\begin{array}{l} J_{\left(E_{1}\right)}=\left(\begin{array}{cccc} -\alpha_{2}T-\mu & -\alpha_{3}S & -\alpha_{1}S & -\alpha_{2}S\\ 0 & \alpha_{3}S-\beta_{2}T-\beta_{3}-\mu & 0 & 0\\ 0 & 0 & \alpha_{1}S-\varepsilon -\gamma_{1}-\mu & 0\\ \alpha_{2}T & \beta_{2}T & \varepsilon & \alpha_{2}S-\gamma_{2}-\mu \end{array}\right) \end{array}$$ (12)

Therefore, the eigenvalues of the matrix *J*_(_*E*__1_)_ are computed as follows:$f\left(\lambda \right)=\lambda^{4}+a_{3}\lambda^{3}+a_{2}\lambda^{2}+a_{1}\lambda +a_{0}$. The coefficient expressions are:


$$\begin{array}{c} \begin{array}{l} a_{3}=\alpha_{2}T+\mu -\left(\alpha_{3}S-\beta_{2}T-\beta_{3}-\mu \right)-\left(\alpha_{1}S-\varepsilon -\gamma_{1}-\mu \right)\\ \;-\left(\alpha_{2}S-\gamma_{2}-\mu \right) \end{array} \end{array}$$

$$\begin{array}{l} \begin{array}{l} a_{2}=\left(-\alpha_{2}T-\mu \right)\left(\alpha_{3}S-\beta_{2}T-\beta_{3}-\mu \right)\\ \;+\left(-\alpha_{2}T-\mu \right)\left(\alpha_{1}S-\varepsilon -\gamma_{1}-\mu \right)\\ \;+\left(-\alpha_{2}T-\mu \right)\left(\alpha_{2}S-\gamma_{2}-\mu \right)\\ \;+\left(\alpha_{3}S-\beta_{2}T_{1}-\beta_{3}-\mu \right)\left(\alpha_{1}S-\varepsilon -\gamma_{1}-\mu \right)\\ \;+\left(\alpha_{3}S-\beta_{2}T-\beta_{3}-\mu \right)\left(\alpha_{2}S-\gamma_{2}-\mu \right)\\ \;+\left(\alpha_{1}S-\varepsilon -\gamma_{1}-\mu \right)\left(\alpha_{2}S-\gamma_{2}-\mu \right)\\ \;+\alpha_{2}^{2}ST \end{array} \end{array}$$

$$\begin{array}{l} \begin{array}{l} a_{1}=-\left(-\alpha_{2}T-\mu \right)-\left(\alpha_{3}S-\beta_{2}T-\beta_{3}-\mu \right)\left(\alpha_{1}S-\varepsilon -\gamma_{1}-\mu \right)\\ \;-\alpha_{2}^{2}ST\left[\left(\alpha_{3}S-\beta_{2}T-\beta_{3}-\mu \right)+\left(\alpha_{1}S-\varepsilon -\gamma_{1}-\mu \right)\right] \end{array} \end{array}$$

$$\begin{array}{l} \begin{array}{l} a_{0}=\left(-\alpha_{2}T-\mu \right)\left(\alpha_{3}S-\beta_{2}T-\beta_{3}-\mu \right)\\ \;\cdot \left(\alpha_{1}S-\varepsilon -\gamma_{1}-\mu \right)\left(\alpha_{2}S-\gamma_{2}-\mu \right)\\ \;-\alpha_{2}^{2}ST\left(\alpha_{3}S-\beta_{2}T-\beta_{3}-\mu \right)\left(\alpha_{1}S-\varepsilon -\gamma_{1}-\mu \right) \end{array} \end{array}$$

According to the Routh-Hurwitz stability criterion, Δ_1_ = *a*_3_, Δ_4_ = *a*_0_Δ_3_, Δ_2_ = *a*_3_*a*_2_−*a*_1_, and $\Delta_{3}=a_{3}a_{2}a_{1}-a_{3}^{2}a_{0}-a_{1}^{2}$. If and *R*_02_ < 1, it can be proved thatΔ_1_>0, Δ_2_>0, Δ_3_>0, and Δ_4_>0. Thus, system (2) is locally asymptotically stable at the infection-free equilibrium point *E*_1_. Using the same method, it can also be proven that the system (2) is locally asymptotically stable at the infection-free equilibrium point *E*_5_.

**(3) The system (2) is locally asymptotically stable at the transmitter-free equilibrium point** ***E***_**2**_.

During rumor propagation, the user population may consist of infected nodes and transmitter nodes. If there are no transmitter nodes willing to publicly state the truth, rumor information can spread more rapidly. Under this scenario, the equilibrium points of the model are denoted as *E*_2_ and *E*_3_. Next, we prove the stability of the equilibrium point *E*_2_.

**Theorem 3** If *R*_01_ < 1, *R*_03_ < 1, and *R*_02_ < 1, then system (2) is locally asymptotically stable at the transmitter-free equilibrium point *E*_2_.

**Proof** The Jacobian matrix of system (2) at the transmitter-free equilibrium point *E*_2_ is given by Equation 13:


(13) $$J_{\left(E_{2}\right)}=\left(\begin{array}{cccc} -\alpha_{1}I-\mu & -\alpha_{3}S & -\alpha_{1}S & -\alpha_{2}S\\ 0 & \alpha_{3}S-\beta_{1}I-\beta_{3}-\mu & 0 & 0\\ \alpha_{1}I & \beta_{1}I & \alpha_{1}S-\varepsilon -\gamma_{1}-\mu & 0\\ 0 & 0 & \varepsilon & \alpha_{2}S-\gamma_{2}-\mu \end{array}\right)$$

Therefore, the eigenvalues of the matrix *J*_(_*E*__2_)_ are computed as follows: $f\left(\lambda \right)=\lambda^{4}+b_{3}\lambda^{3}+b_{2}\lambda^{2}+b_{1}\lambda +b_{0}$. The coefficient expressions are:


$$\begin{array}{l} b_{3}=\alpha_{1}I+\mu -\alpha_{3}S+\beta_{1}I+\beta_{3}+\mu -\alpha_{1}S+\varepsilon +\gamma_{1}+\mu )\\ \;-\alpha_{2}S+\gamma_{2}+\mu \end{array}$$

$$\begin{array}{l} b_{2}\;=(-\alpha_{1}I-\mu )(\alpha_{3}S-\beta_{1}I-\beta_{3}-\mu )\\ \;+[(-\alpha_{1}I-\mu )(\alpha_{1}S-\varepsilon -\gamma_{1}-\mu )+\alpha_{1}^{2}IS]\\ \;+(-\alpha_{1}I-\mu )(\alpha_{2}S-\gamma_{2}-\mu )\\ \;+(\alpha_{3}S-\beta_{1}I-\beta_{3}-\mu )(\alpha_{1}S_{1}-\varepsilon -\gamma_{1}-\mu )\\ \;+(\alpha_{3}S-\beta_{1}I-\beta_{3}-\mu )(\alpha_{2}S-\gamma_{2}-\mu )\\ \;+(\alpha_{1}S-\varepsilon -\gamma_{1}-\mu )(\alpha_{2}S-\gamma_{2}-\mu )\\ \;+\alpha_{2}^{2}S_{1}T_{1} \end{array}$$

$$\begin{array}{l} b_{1}=-[(\alpha_{3}S-\beta_{1}I-\beta_{3}-\mu )\cdot (\alpha_{1}S-\varepsilon -\gamma_{1}-\mu )\\ \;(\alpha_{2}S-\gamma_{2}-\mu )+(-\alpha_{1}I-\mu )(\alpha_{1}S-\varepsilon -\gamma_{1}-\mu )\\ \;+(\alpha_{2}S-\gamma_{2}-\mu )+\alpha_{1}^{2}IS(\alpha_{2}S-\gamma_{2}-\mu )-\alpha_{1}\alpha_{2}\\ \;IS+(-\alpha_{1}I-\mu )(\alpha_{3}S-\beta_{1}I-\beta_{3}-\mu )(\alpha_{2}S-\gamma_{2}-\mu )+\\ \;(-\alpha_{1}I-\mu )(\alpha_{3}S-\beta_{1}I-\beta_{3}-\mu )(\alpha_{1}S-\varepsilon -\gamma_{1}-\mu )\\ \;+(\alpha_{3}S-\beta_{1}I-\beta_{3}-\mu )\alpha_{1}^{2}IS] \end{array}$$

$$\begin{array}{l} b_{0}\;=(\alpha_{3}S-\beta_{1}I-\beta_{3}-\mu )\\ \;\cdot [(-\alpha_{1}I-\mu )(\alpha_{1}S-\varepsilon -\gamma_{1}-\mu )(\alpha_{2}S-\gamma_{2}-\mu )\\ \;+\alpha_{1}^{2}IS(\alpha_{2}S-\gamma_{2}-\mu )-\alpha_{1}\alpha_{2}IS] \end{array}$$

According to the Routh-Hurwitz stability criterion Δ_1_ = *b*_3_, Δ_4_ = *b*_0_Δ_3_, Δ_2_ = *b*_3_*b*_2_−*b*_1_, and $\Delta_{3}=b_{3}\left(b_{2}b_{1}-b_{3}b_{0}\right)-b_{1}^{2}$. if *R*_01_ < 1, *R*_03_ < 1, *R*_02_>1, it can be proven that Δ_1_>0, Δ_2_>0, Δ_3_>0, Δ_4_>0. Thus, system (2) is locally asymptotically stable at the rumor equilibrium point *E*_2_. Using the same method, it can also be proven that system (2) is locally asymptotically stable at the rumor equilibrium point *E*_3_.

**(4) System (2) is locally asymptotically stable at the coexistence equilibrium point** ***E***_4_, **where both infected nodes and transmitter nodes are present**.

During rumor propagation, the user population may consist of infected nodes and transmitter nodes. When both infected nodes and transmitter nodes coexist, the corresponding equilibrium points for the infected and transmitter nodes are *E*_4_ and *E*_6_. The following is a proof of the stability of equilibrium point *E*_4_:

**Theorem 4** If *R*_01_ > 1, *R*_02_ > 1, and *R*_03_ < 1, then system (2) is locally asymptotically stable at the coexistence equilibrium point *E*_4_, where both infected nodes and transmitter nodes are present.

**Proof** The Jacobian matrix of system (2) at the transmitter-free equilibrium point *E*_4_ is given by Equation 14:


$$\begin{array}{l} \;J_{\left(E_{4}\right)}=\\ \left(\begin{array}{cccc} -\alpha_{1}I-\alpha_{2}T-\mu & -\alpha_{3}S & -\alpha_{1}S & -\alpha_{2}S\\ 0 & \alpha_{3}S-\beta_{1}I-\beta_{2}T-\beta_{3}-\mu & \beta_{1}I & \beta_{2}T\\ \alpha_{1}I & 0 & \alpha_{1}S-\varepsilon -\gamma_{1}-\mu & \varepsilon \\ \alpha_{2}T & 0 & 0 & \alpha_{2}S-\gamma_{2}-\mu \end{array}\right) \end{array}$$ (14)

Therefore, the eigenvalues of the matrix *J*_(_*E*__4_)_ are computed as follows: $f\left(\lambda \right)=\lambda^{4}+c_{3}\lambda^{3}+c_{2}\lambda^{2}+c_{1}\lambda +c_{0}$. The coefficient expressions are:


$$\begin{array}{l} c_{3}=\left(\alpha_{1}I+\alpha_{2}T+\mu \right)-\left(\alpha_{3}S-\beta_{1}I-\beta_{2}T-\beta_{3}-\mu \right)\\ \;-\left(\alpha_{1}S-\varepsilon -\gamma_{1}-\mu \right)-\left(\alpha_{2}S-\gamma_{2}-\mu \right) \end{array}$$

$$\begin{array}{l} c_{2}=(-\alpha_{1}I-\alpha_{2}T-\mu )(\alpha_{3}S-\beta_{1}I-\beta_{2}T-\beta_{3}-\mu )\\ \;+(-\alpha_{1}I-\alpha_{2}T-\mu )(\alpha_{1}S-\varepsilon -\gamma_{1}-\mu )\\ \;+(-\alpha_{1}I-\alpha_{2}T-\mu )(\alpha_{2}S-\gamma_{2}-\mu )\\ \;+(\alpha_{3}S-\beta_{1}I-\beta_{2}T-\beta_{3}-\mu )(\alpha_{1}S-\varepsilon -\gamma_{1}-\mu )\\ \;+(\alpha_{3}S-\beta_{2}T_{1}-\beta_{3}-\mu )(\alpha_{2}S-\gamma_{2}-\mu )\\ \;+(\alpha_{1}S-\varepsilon -\gamma_{1}-\mu )(\alpha_{2}S-\gamma_{2}-\mu )\\ \;+\alpha_{1}\alpha_{4}IS+\alpha_{2}^{2}ST \end{array}$$

$$\begin{array}{l} c_{1}=(\alpha_{1}I+\alpha_{2}T+\mu )[(\alpha_{3}S-\beta_{1}I-\beta_{2}T-\beta_{3}-\mu )\\ \left(\alpha_{1}S-\varepsilon -\gamma_{1}-\mu )](\alpha_{3}S-\beta_{1}I-\beta_{2}T-\beta_{3}-\mu )(\alpha_{2}S-\gamma_{2}-\mu \right)\\ +(\alpha_{1}S-\varepsilon -\gamma_{1}-\mu )(\alpha_{2}S-\gamma_{2}-\mu )\\ -(\alpha_{3}S-\beta_{1}I-\beta_{2}T-\beta_{3}-\mu )(\alpha_{1}S-\varepsilon -\gamma_{1}-\mu )(\alpha_{2}S-\gamma_{2}-\mu )\\ -\alpha_{1}^{2}IS(\alpha_{2}S-\gamma_{2}-\mu )+\alpha_{1}\alpha_{2}\varepsilon ST-\alpha_{2}^{2}(\alpha_{1}S-\varepsilon -\gamma_{1}-\mu )ST\\ +\alpha_{2}\alpha_{3}\beta_{2}ST^{2}-\alpha_{2}^{2}(\alpha_{3}S-\beta_{1}I-\beta_{2}T-\beta_{3}-\mu )ST+\alpha_{1}\alpha_{3}\beta_{1}SI^{2}\\ -\alpha_{1}^{2}(\alpha_{3}S-\beta_{1}I-\beta_{2}T-\beta_{3}-\mu )SI \end{array}$$

$$\begin{array}{l} c_{0}=\;(\alpha_{3}S-\beta_{1}I-\beta_{2}T-\beta_{3}-\mu )[-\alpha_{2}\alpha_{4}TS+\alpha_{2}^{2}TS\\ \;(\alpha_{1}S-\varepsilon -\gamma_{1}-\mu )+(-\alpha_{1}I-\alpha_{2}T-\mu )(\alpha_{1}S-\varepsilon -\gamma_{1}-\mu )\cdot \\ \;(\alpha_{2}S-\gamma_{2}-\mu )+(\alpha_{2}S-\gamma_{2}-\mu )\alpha_{1}\alpha_{4}IS]\\ \;-\alpha_{1}\alpha_{3}\beta_{1}I^{2}S(\alpha_{2}S-\gamma_{2}-\mu )+\alpha_{2}\alpha_{3}\beta_{1}IST-\alpha_{2}\alpha_{3}\beta_{2}ST^{2}\\ \;(\alpha_{1}S-\varepsilon -\gamma_{1}-\mu ) \end{array}$$

According to the Routh-Hurwitz stability criterionΔ_1_ = *c*_3_, Δ_2_ = *c*_3_*c*_2_−*c*_1_, $\Delta_{3}=c_{3}c_{2}c_{1}-c_{3}^{2}c_{0}-c_{1}^{2}$, and Δ_4_ = *c*_0_Δ_3_, if *R*_01_>1, *R*_02_>1, *R*_03_ < 1, it can be proven that Δ_1_>0, Δ_2_>0, Δ_3_>0, Δ_4_>0. Therefore, system (2) is locally asymptotically stable at the coexistence equilibrium point *E*_4_ where both infected nodes and transmitter nodes are present. Similarly, using the same method, it can also be proven that system (2) is locally asymptotically stable at the rumor equilibrium point *E*_6_.

### 2.5 Global asymptotic stability of equilibrium points

Global asymptotic stability refers to a type of asymptotic stability where all regions in the phase space serve as basins of attraction. In the study, the second method of Lyapunov is employed to prove that the rumor-free equilibrium point *E*_0_ possesses global asymptotic stability.

**Theorem 5** If *R*_01_ < 1, *R*_02_ < 1, and *R*_03_ < 1, then the rumor-free equilibrium point of system (2) is globally asymptotically stable.

**Proof** Construct a Lyapunov function as shown in Equation 15:


$$\begin{array}{l} V\left(I\left(t\right),S\left(t\right)\right)=\frac{1}{2}\left(k_{0}I{\left(t\right)}^{2}+k_{1}S{\left(t\right)}^{2}\right) \end{array}$$ (15)

Where both *k*_0_ and *k*_1_ are positive real numbers.

Since the constructed Lyapunov function satisfies the positive definiteness condition, we proceed to compute its time derivative along system (2). The calculation formula is shown in (16):


$$\begin{array}{l} \frac{dV}{dt}\;=\;(\alpha_{1}SI+\beta_{1}NI-\varepsilon I-\gamma_{1}I-\mu I)\\ \;+(\alpha_{3}SN-\beta_{1}IN-\beta_{2}TN-\beta_{3}N-\mu N)\\ \;=\alpha_{1}SI-\varepsilon I-\gamma_{1}I-\mu I+\alpha_{3}SN-\beta_{3}N-\mu N+\beta_{1}NI\\ \;+\beta_{1}IN-\beta_{2}TN) \end{array}$$ (16)

Since β_1_*NI*+β_1_*IN* = 0, Equation 16 can be simplified as shown in Equation 17:


$$\begin{array}{l} \frac{dV}{dt}=I\left(\alpha_{1}S-\varepsilon -\gamma_{1}-\mu \right)+N\left(\alpha_{3}S-\beta_{3}-\mu \right)-\beta_{2}TN \end{array}$$ (17)

Since *S*(*t*) ≤ 1, we have α_1_*S* ≤ α_1_, therefore:


$$\begin{array}{l} \alpha_{1}S-\varepsilon -\gamma_{1}-\mu \leq \alpha_{1}-\varepsilon -\gamma_{1}-\mu =-\left(\varepsilon +\gamma_{1}+\mu \right)\\ \;(1-R_{01})<0 \end{array}$$

Since *R*_01_ < 1, let δ = (ε+γ_1_+μ)(1−*R*_01_)>0, therefore: *I*(α_1_*S*−ε−γ_1_−μ) ≤ −δ*I* ≤ 0.

Since α_3_*S* ≤ α_3_, at the same timeα_3_−β_3_−μ < 0, therefore: *N*(α_3_*S*−β_3_−μ) ≤ *N*(α_3_−β_3_−μ) < 0.

Thus, it is proven that all terms are non-positive.

When*I* = 0 and *N* = 0, system (2) simplifies to:$\frac{dS}{dt}=\mu -\mu S$,$\frac{dT}{dt}=\alpha_{2}ST-\gamma_{2}T-\mu T$.

Assumingα_2_−γ_2_−μ < 0, therefore,$\frac{dT}{dt}\leq \left(\alpha_{2}-\gamma_{2}-\mu \right)T<0$. If *T*>0, then*T* → 0.

Meanwhile, $\frac{dS}{dt}=\mu \left(1-S\right)$, therefore, *S* → 1, Consequently, the system converges to(1, 0, 0, 0). According to LaSalle's invariance principle, when*I*(*t*) → ∞ or *S*(*t*) → ∞ is satisfied,*V*(*I*(*t*), *S*(*t*)) → ∞, therefore, the proof of Theorem 5 is completed.

## 3 System simulation and result analysis

### 3.1 Local asymptotic stability analysis of equilibrium points

**Figure 3:**
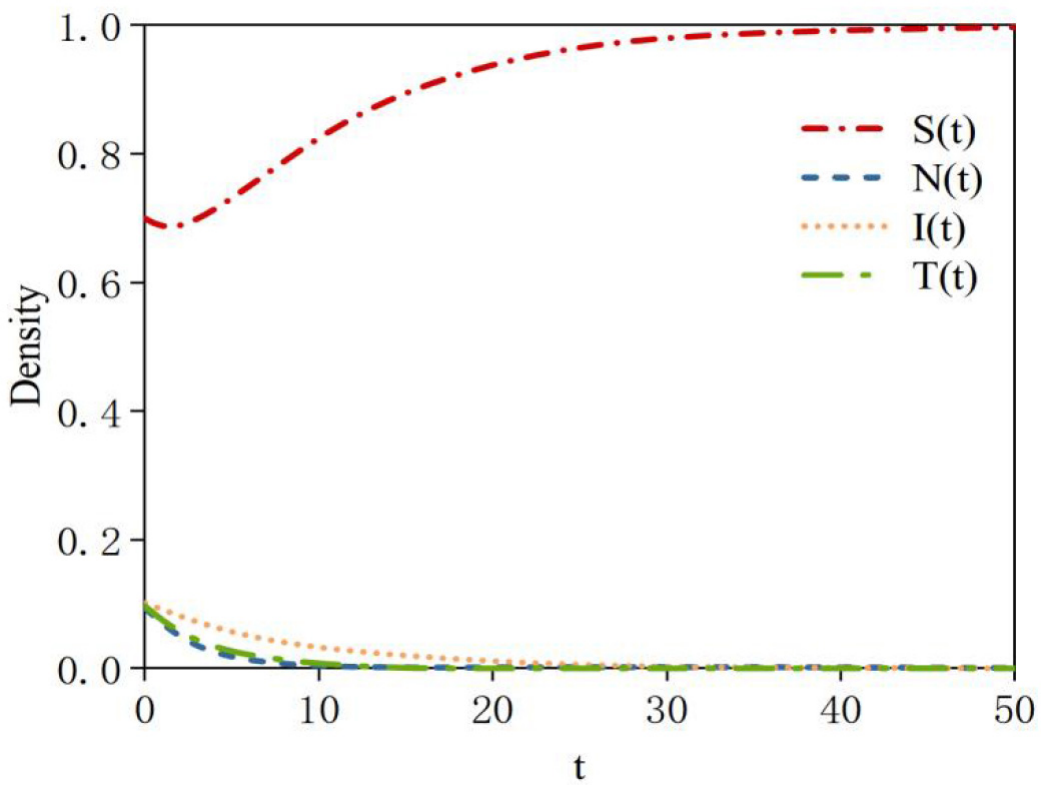
Density evolution of the four states at equilibrium point *E0*.

First, it is necessary to verify that the rumor-free equilibrium point *E*_0_ is locally asymptotically stable. According to Theorem 1, system (2) is locally asymptotically stable at the equilibrium point *E*_0_. Based on the data in Table 2, the density curves of susceptible, neglected, infected, and transmitter nodes at the rumor-free equilibrium point *E*_0_ can be obtained. The density evolution is shown in Figure 3. In the initial phase of the system, when susceptible nodes come into contact with rumors, some are unable to identify the rumors and quickly transform into neglected or infected nodes, while others recognize the rumors and become transmitter nodes. As a result, the density of susceptible nodes decreases. Subsequently, as rumor dissemination gradually ceases within the system and new nodes no longer encounter rumors, the density of susceptible nodes rebounds to 1 and eventually stabilizes. Meanwhile, due to the lack of sustained interactive momentum, the densities of neglected nodes, infected nodes, and transmitter nodes gradually diminish to zero.

**Table 2:** Parameters for local asymptotic stability of equilibrium point *E0*.

Parameters	α_1_	α_2_	α_3_	μ	β_1_	β_2_	β_3_	ε	γ_1_	γ_2_
Date	0.25	0.35	0.28	0.28	0.3	0.35	0.3	0.3	0.3	0.3

During the rumor propagation process, the excessive influence of infected nodes may lead to the absence of transmitter nodes on a social network. In this case, the equilibrium points are *E*_2_ and *E*_3_. According to Theorem 3, system (2) is locally asymptotically stable at equilibrium points *E*_2_ and *E*_3_.

**Figure 4:**
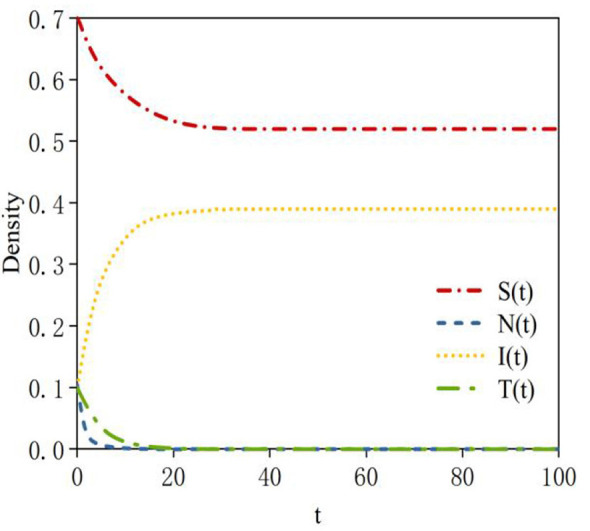
Density evolution of the four states at equilibrium point *E*_2_.

Based on Data 1 in Table 3, the density evolution process of nodes at equilibrium point *E*_2_ can be obtained, as shown in Figure 4. Here, the basic reproduction number is *R*_01_ = 0.27 < 1; this indicates that the probability of susceptible nodes transforming into infected nodes is low. *R*_02_ = 1.77 >1 indicates that the probability of neglected nodes transforming into infected nodes is high; *R*_03_ = 0.75 < 1 indicates that the sustained propagation capability of infected nodes is relatively weak. When system (2) reaches a steady state, a rumor-persistent equilibrium point, E_2_ = (0.51, 0, 0.39, 0), exists. In this case, the probability of susceptible nodes transforming into infected nodes is low; therefore, the density of susceptible nodes is high at equilibrium. All neglected nodes transform into infected nodes; thus, the density of infected nodes is relatively high at equilibrium. The sustained propagation capability of infected nodes is relatively weak.

**Table 3:** Parameters for local asymptotic stability of equilibrium points *E*_2_ and *E3*.

Parameters	α_1_	α_2_	α_3_	μ	β_1_	β_2_	β_3_	ε	γ_1_	γ_2_
Date 1	0.2	0.46	0.25	0.18	0.25	0.3	0.15	0.4	0.15	0.05
Date 2	0.6	0.18	0.18	0.18	0.5	0.05	0.15	0	0.05	0.15

**Figure 5:**
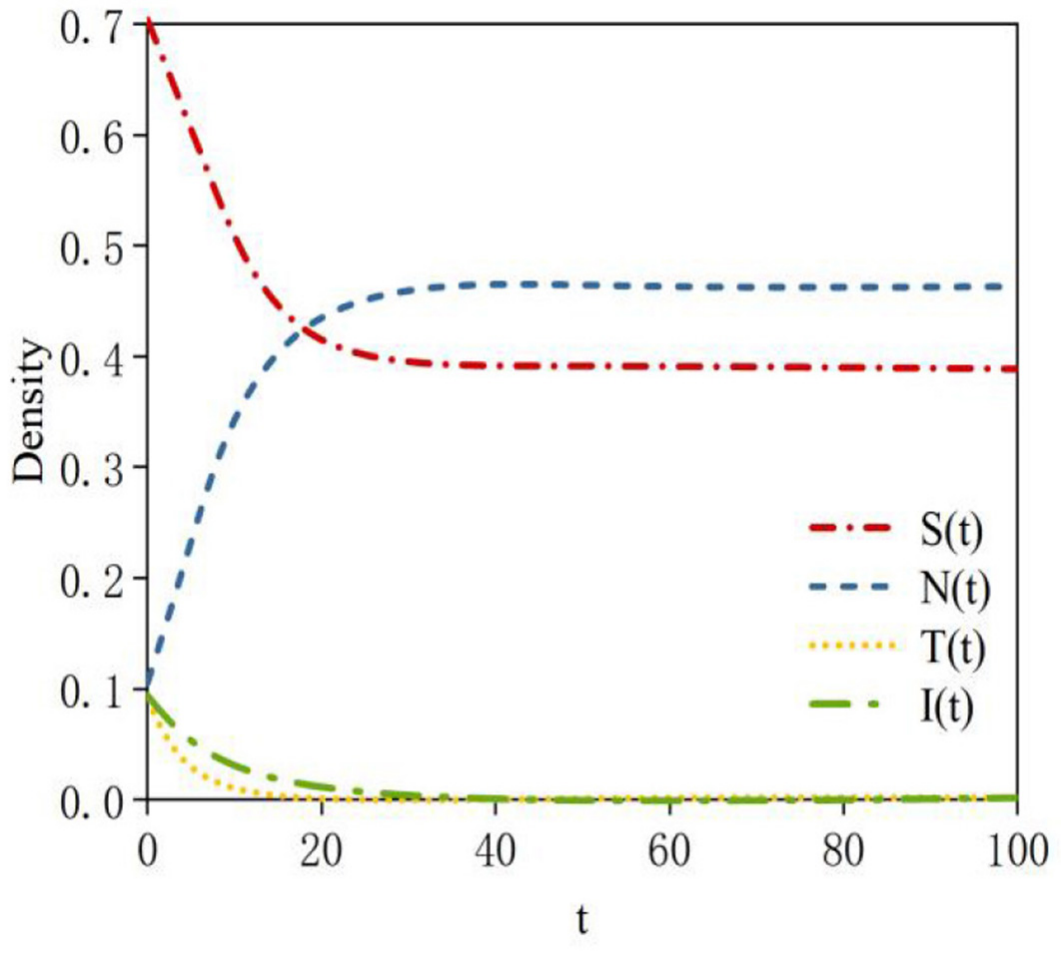
Density evolution of the four states at equilibrium point *E3*.

According to Data 2 in Table 3, the density evolution process of nodes at equilibrium point *E3* can be obtained, as shown in Figure 5. Here, the basic reproduction number is *R*_01_ = 2.6 >1; this indicates that the probability of susceptible nodes transforming into infected nodes is high. *R*_02_ = 0.61 < 1 indicates that the probability of neglected nodes transforming into infected nodes is low; *R*_03_ = 0.61 < 1 indicates that the sustained propagation capability of infected nodes is relatively weak. When system (2) reaches a steady state, a rumor-persistent equilibrium point, *E*_3_ = (0.38, 0.47, 0, 0), exists. In this case, the probability of susceptible nodes transforming into infected nodes is relatively high; therefore, the density of susceptible nodes at equilibrium decreases compared to equilibrium point *E*_2_. The probability of neglected nodes transforming into infected nodes is relatively low; thus, the density of neglected nodes at equilibrium is relatively high. The sustained propagation capability of infected nodes is relatively weak; consequently, the density of infected nodes is zero at equilibrium.

**Figure 6:**
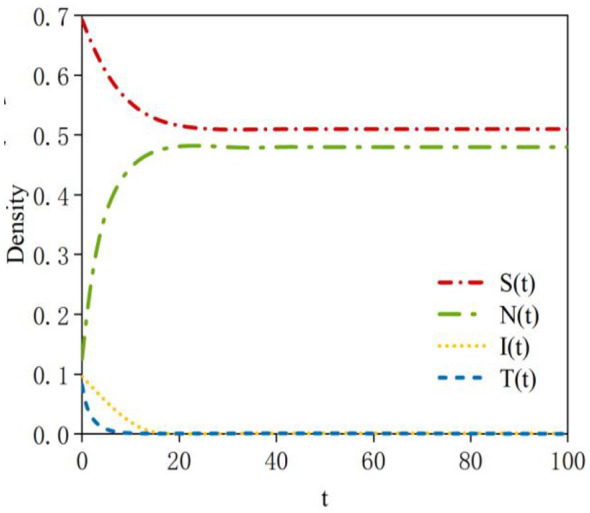
Density evolution of the four states at equilibrium point *E*_1._

During the rumor propagation process, due to the continuous refutation efforts of transmitter nodes, the number of infected nodes on a social network gradually decreases until it reaches zero. In this case, the equilibrium points are *E1* and *E*_5_. According to Theorem 2, system (2) is locally asymptotically stable at equilibrium points *E1* and *E*_5_. Based on Data 1 in Table 4, the density evolution of nodes at equilibrium point *E*1 can be obtained, as shown in Figure 6. Here, the basic reproduction number satisfies *R*_01_ = 0.57 < 1; this indicates that the probability of susceptible nodes transforming into infected nodes is low. *R*_02_ = 0.76 < 1 indicates that the probability of neglected nodes transforming into infected nodes is low; *R*_03_1 = 1.45 >1 indicates that the sustained propagation capability of infected nodes is relatively strong. When system (2) is in a steady state, a rumor-free equilibrium point, *E*_1_ = (0.51, 0, 0, 0.48), exists. In this case, the probability of susceptible nodes transforming into infected nodes is relatively low, therefore the density of susceptible nodes is relatively high at equilibrium; the probability of neglected nodes transforming into infected nodes is relatively low, while the sustained propagation capability of transmitter nodes is relatively strong, therefore, the densities of neglected nodes and infected nodes eventually drop to zero.

**Table 4:** Parameters for local asymptotic stability of equilibrium points *E1* and *E*5.

Parameters	α_1_	α_2_	α_3_	μ	β_1_	β_2_	β_3_	ε	γ_1_	γ_2_
Date 1	0.3	0.25	0.38	0.18	0.3	0.3	0.05	0.2	0.15	0.15
Date 2	0.5	0.24	0.24	0.18	0.45	0.2	0.05	0.08	0.05	0.15

**Figure 7:**
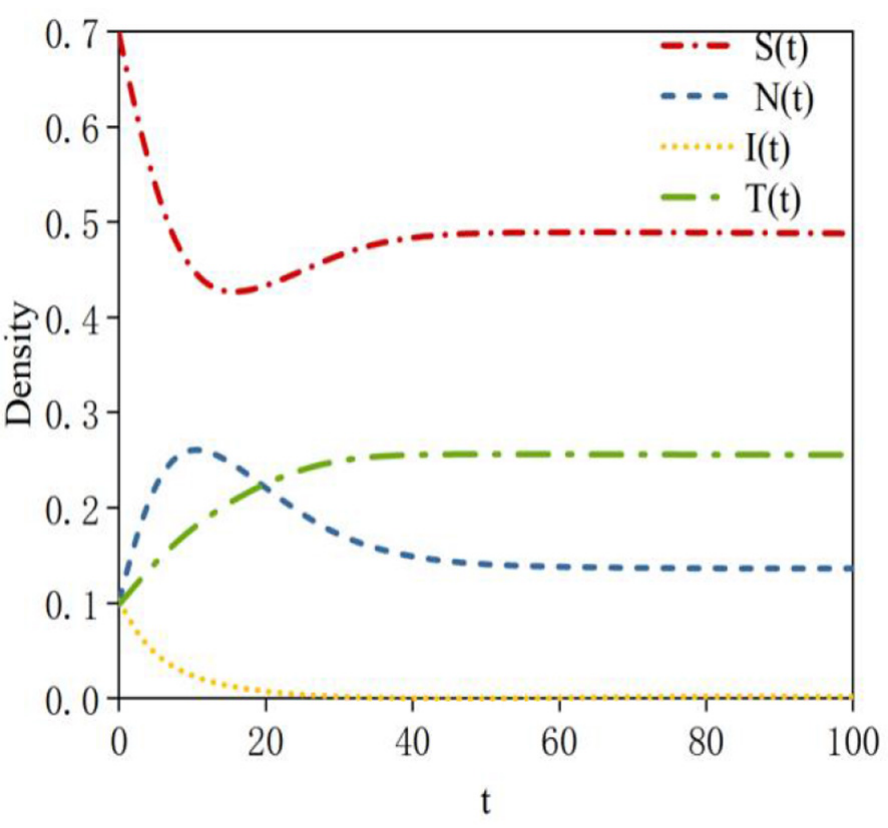
Density evolution of the four states at equilibrium point *E*_5._

According to Data 2 in Table 4, the density evolution of nodes at equilibrium point *E*_5_ can be obtained, as shown in Figure 7. Here, the basic reproduction number satisfies *R*_01_ = 2.3 >1; this indicates that the probability of susceptible nodes transforming into infected nodes is high. *R*_02_ = 0.91 < 1 indicates that the probability of neglected nodes transforming into infected nodes is low; *R*_03_ = 1.48 >1 indicates that the sustained propagation capability of infected nodes is relatively strong. When system (2) is in a steady state, a rumor-free equilibrium point, *E*_5_ = (0.48, 0.14, 0, 0.24), exists. In this case, the probability of susceptible nodes transforming into infected nodes is relatively high, leading to a decrease in the density of susceptible nodes compared to equilibrium point *E*1. The probability of neglected nodes transforming into infected nodes is relatively low, the sustained propagation capability of transmitter nodes is relatively strong. Consequently, the density of infected nodes is zero at equilibrium.

During the rumor propagation process, it is common for both infected nodes and transmitter nodes to coexist. In this case, the equilibrium points are *E*_4_ and *E*_6_. According to Theorem 4, system (2) is locally asymptotically stable at equilibrium points *E*_4_ and *E*_6_.

**Figure 8:**
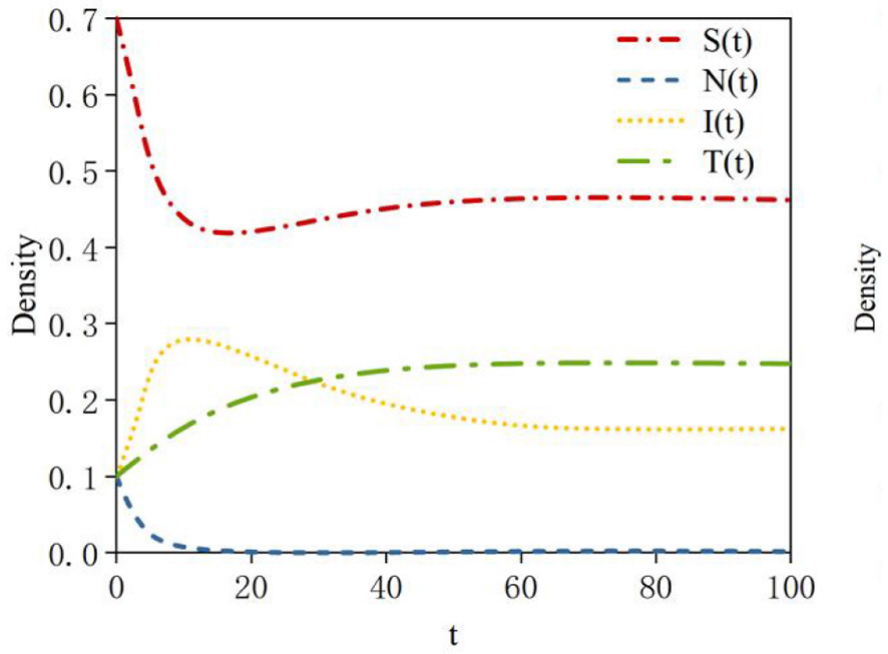
Density evolution of the four states at equilibrium point *E*_4._

Based on Data 1 in Table 5, the density evolution of nodes at equilibrium point *E*_4_ can be obtained, as shown in Figure 8. Here, the basic reproduction number satisfies *R*_01_ = 2.3 >1; this indicates that the probability of susceptible nodes transforming into infected nodes is high. *R*_02_ = 2.87 >1 indicates that the probability of neglected nodes transforming into infected nodes is high; *R*_03_ = 1.65 >1 indicates that the sustained propagation capability of infected nodes is relatively strong. When system (2) is in a steady state, a rumor-persistent equilibrium point *E*_4_ = (0.44, 0, 0.16, 0.24) exists. In this case, transmitter nodes are present. The probability of susceptible nodes transforming into infected nodes is relatively high, resulting in a lower density of susceptible nodes. This indicates that the probability of neglected nodes transforming into infected nodes is also relatively high, while the sustained propagation capability of infected nodes remains strong. Consequently, the density of neglected nodes eventually drops to zero, and the density of infected nodes remains relatively low.

**Table 5:** Parameters for local asymptotic stability of equilibrium points *E4* and *E6*.

Parameters	α_1_	α_2_	α_3_	μ	β_1_	β_2_	β_3_	ε	γ_1_	γ_2_
Date 1	0.2	0.36	0.38	0.18	0.2	0.32	0.05	0.08	0.15	0.05
Date 2	0.46	0.25	0.17	0.18	0.25	0.35	0.05	0.08	0.05	0.05

**Figure 9:**
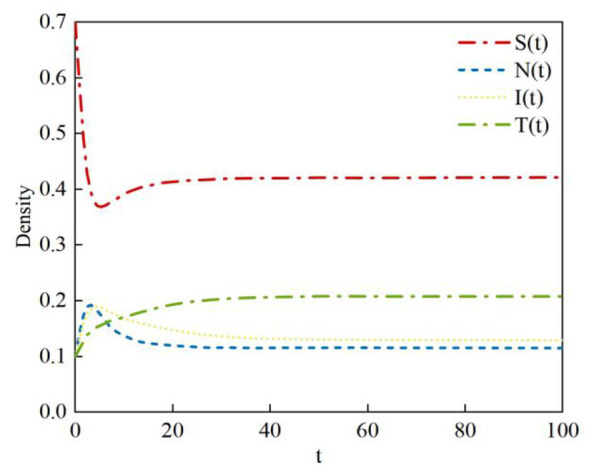
Density evolution of the four states at equilibrium point *E*_6._

According to Data 2 in Table 5, the density evolution of nodes at equilibrium point *E*_6_ can be obtained, as shown in Figure 9. Here, the basic reproduction number is *R*_01_ = 2.77 >1, this indicates that the probability of susceptible nodes transforming into infected nodes is high. *R*_02_ = 2.39>1 indicates that the probability of neglected nodes transforming into infected nodes is high; *R*_03_ = 1.61>1 indicates that the sustained propagation capability of infected nodes is relatively strong. When system (2) reaches a steady state, a rumor-persistent equilibrium point, *E*_6_ = (0.42, 0.12, 0.13, 0.21), exists. In this case, due to varying attitudes of network nodes toward the rumor after exposure, neglected nodes, infected nodes, and transmitter nodes all coexist. Regardless of how the initial values change, the density of rumor-spreading nodes eventually stabilizes and reaches the equilibrium point.

### 3.2 Impact of key parameters on infected nodes

**Figure 10:**
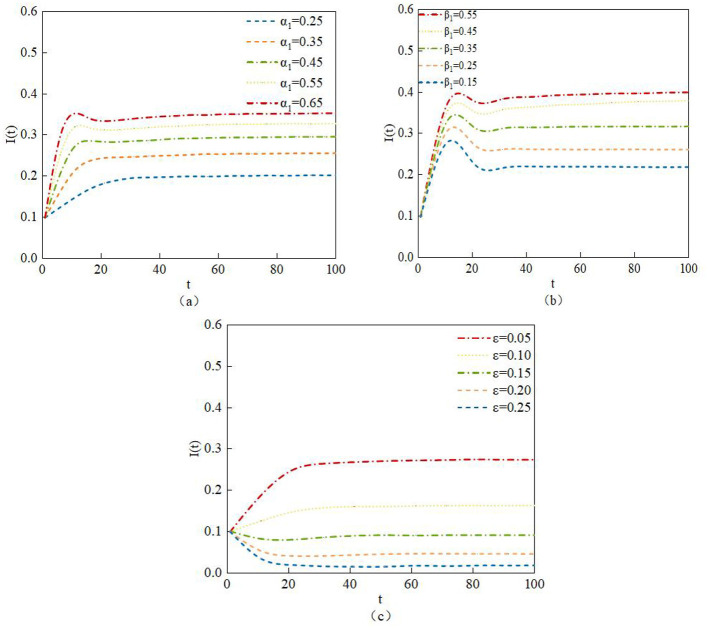
Density evolution of I(t) under variations in parameters **(a)** α*1*, **(b)** β1, and **(c)** ε.

Determined by system (2), the density of infected nodes is jointly influenced by the infection proportion α1, the infection proportion β1, and the infection proportion ε. The study analyzes the impact of different values of α1, β1, and ε on the density of infected nodes through numerical experiments. Figure 10 shows the dynamic evolution curves of infected node density under different values of α1, β1, and ε, with the corresponding data values listed as Data 1 to Data 3 in Table 6.

**Table 6:** Parameter values affecting infected nodes I.

Parameters	α_1_	α_2_	α_3_	μ	β_1_	β_2_	β_3_	ε	γ_1_	γ_2_
Date 1	[0.25–0.65]	0.15	0.15	0.18	0.25	0.35	0.05	0.08	0.05	0.05
Date 2	0.55	0.2	0.15	0.18	[0.15–0.55]	0.35	0.05	0.05	0.05	0.05
Date 3	0.55	0.2	0.15	0.18	0.25	0.35	0.05	[0.05–0.25]	0.05	0.05

The simulation results in Figures 10a, b show that, as the infection proportion α*1* and β*1* increase over time, more susceptible nodes and neglected nodes transform into infected nodes; the density of infected nodes I also correspondingly increases. From Figure 10c, it can be observed that, as the infection proportion ε increases, more infected nodes transform into transmitter nodes; the density of infected nodes I correspondingly decreases. Therefore, appropriately reducing the values of the infection proportion α*1* and β*1* while increasing the value of infection proportion ε, can reduce the number of infected nodes I and suppress the spread of rumors in the system, which aligns with the characteristics of rumor propagation.

According to system (2), the density of transmitter nodes is closely related to the infection proportion α_2_ and β_2_ and the infection proportion ε. The study investigates the impact of different values of α_2_, β_2_, and ε on the density of transmitter nodes. The corresponding data values are listed as Data 1 to Data 3 in Table 7.

**Table 7:** Influence of the transmitter node T parameter on its value setting.

Parameters	α_1_	α_2_	α_3_	μ	β_1_	β_2_	β_3_	ε	γ_1_	γ_2_
Date 1	0.15	[0.2–0.6]	0.15	0.18	0.25	0.35	0.05	0.08	0.05	0.05
Date 2	0.3	0.66	0.38	0.18	0.2	[0.15–0.35]	0.05	0.08	0.15	0.05
Date 3	0.7	0.35	0.2	0.18	0.4	0.2	0.15	[0.05–0.13]	0.05	0.05

**Figure 11:**
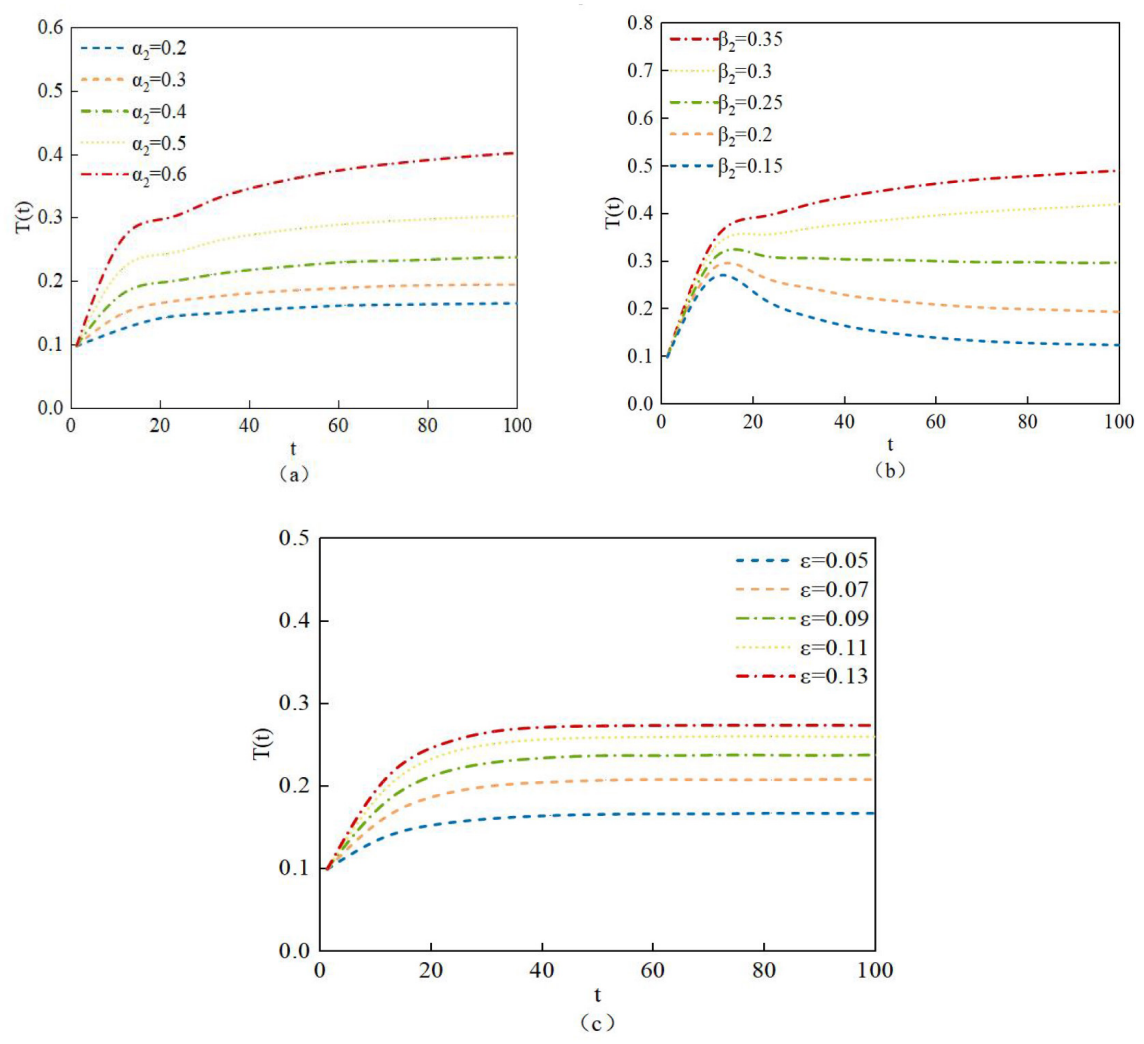
Changes in the density evolution of T(t) with different parameter values of **(a)** α_2_, **(b)** β_2_, and **(c)** ε.

Figure 11 shows the evolutionary curves of the density of transmitter nodes under different proportions of α_2_, β_2_, and ε. From Figures 11a–c, it can be observed that, as the infection proportion α_2_, infection proportion β_2_, and infection conversion proportion ε increase, more susceptible nodes, neglected nodes, and infected nodes transform into transmitter nodes, therefore, the density of transmitter nodes T also increases accordingly. Therefore, appropriately raising the values of the infection proportion α_2_, infection proportion β_2_, and infection conversion proportion ε can increase the number of transmitter nodes, thereby enabling nodes that receive rumors to better identify them and reducing the likelihood of nodes being influenced by rumors.

### 3.3 Sensitivity analysis of the basic reproduction number

The study conducts a sensitivity analysis of the basic reproduction number to investigate the influence of different parameters on it.

**Figure 12:**
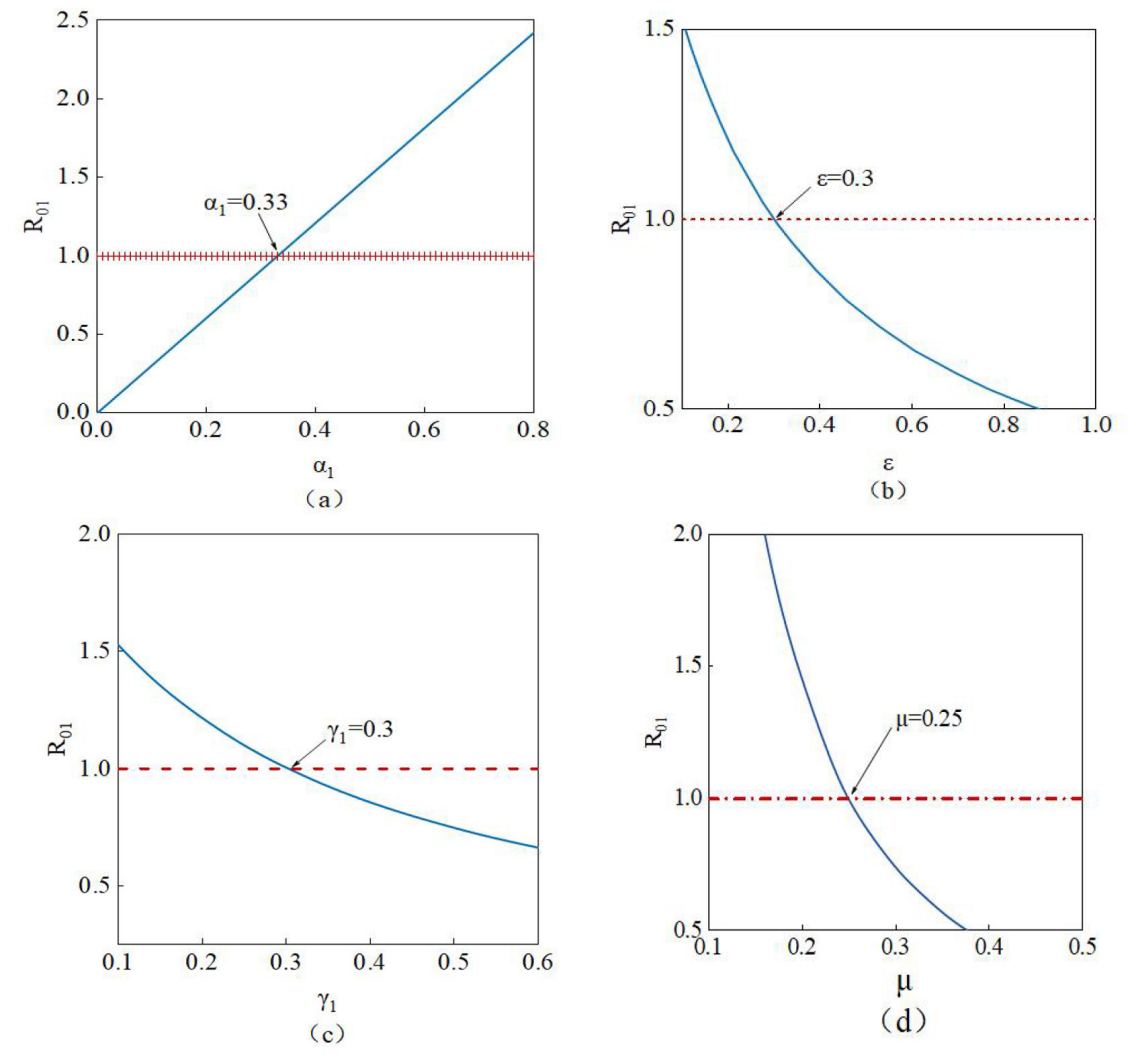
Impact of parameters **(a)** α*1*, **(b)** ε, **(c)** γ*1*, and **(d)** μ on *R01*.

$R_{01}=\frac{\alpha_{1}}{\varepsilon +\gamma_{1}+\mu }$, The parameters involved *R*_01_are α*1*, ε, γ*1*, and μ. From Figures 12a–d, it can be observed that the value of *R*01 increases as the parameter α*1* increases. Therefore, as the value of α*1* rises, susceptible nodes become more likely to trust the rumor, increasing the probability of their transformation into infected nodes. Conversely, the value of *R*01 decreases as the parameters ε, γ*1*, and μ increase. Thus, higher values of ε, γ*1*, and μ lead to a reduction in *R*01 until it falls below 1, indicating effective suppression of the rumor. In summary, when *R*01 < 1, the rumor will be controlled.

**Figure 13:**
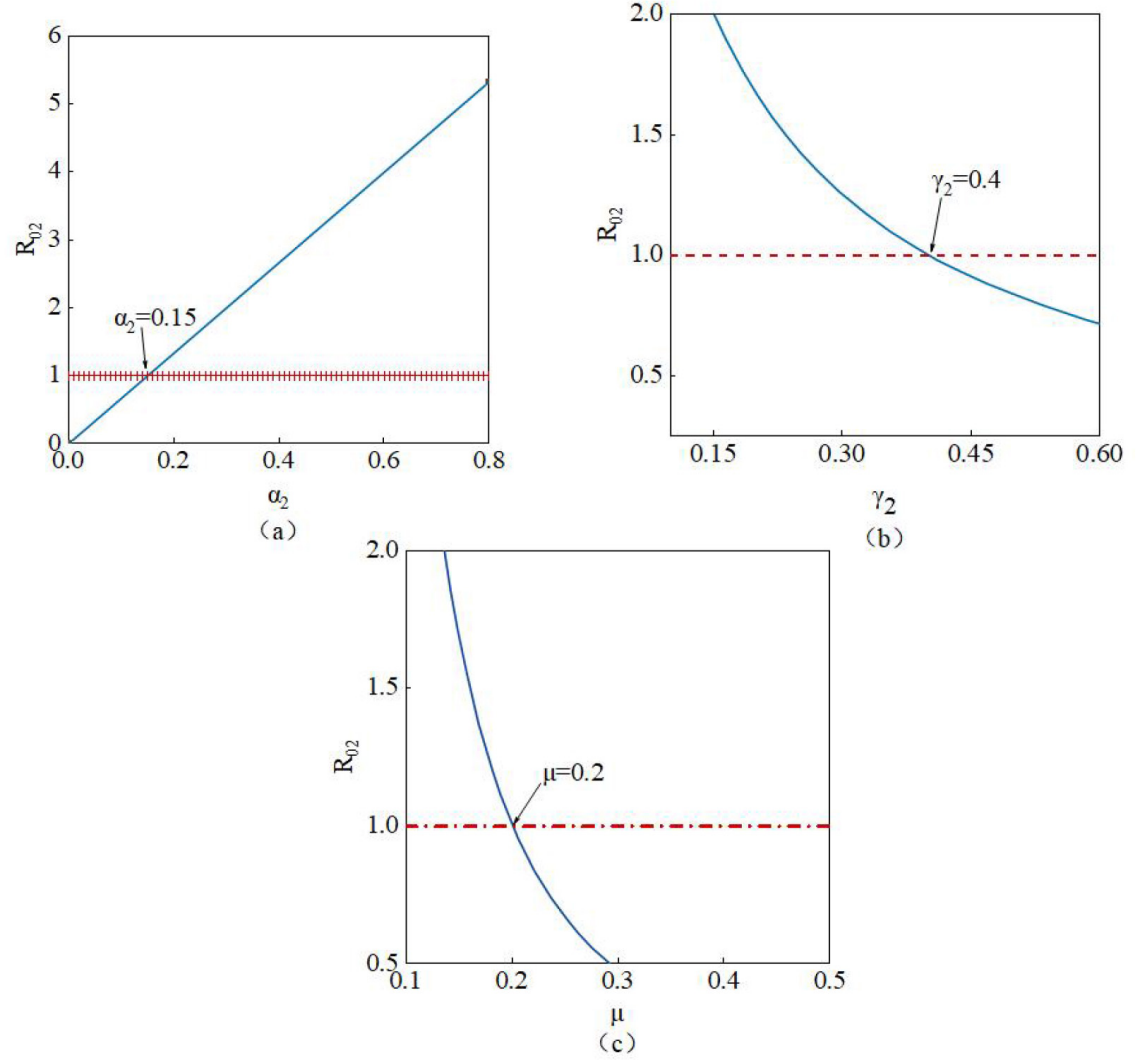
Impact of parameters **(a)** α_2_, **(b)** γ_2_, and **(c)** μ on *R0*_2_.

$R_{02}=\frac{\alpha_{2}}{\gamma_{2}+\mu }$, The parameters involved in *R*_02_ are α_2_, γ_2_, and μ. From Figures 13 a–c, it can be observed that the value of *R*0_2_ increases as the parameter α_2_ increases. Therefore, a larger value of α_2_ indicates that susceptible nodes are more likely to believe the rumor and transform into infected nodes. Conversely, the value of *R*0_2_ decreases as the parameters γ_2_ and μ increase. Thus, higher values of γ_2_ and μ lead to a reduction in *R*0_2_ until it falls below 1, thereby effectively suppressing the spread of the rumor. In summary, when *R*0_2_ < 1, the spread of the rumor will be controlled and eventually cease.

**Figure 14:**
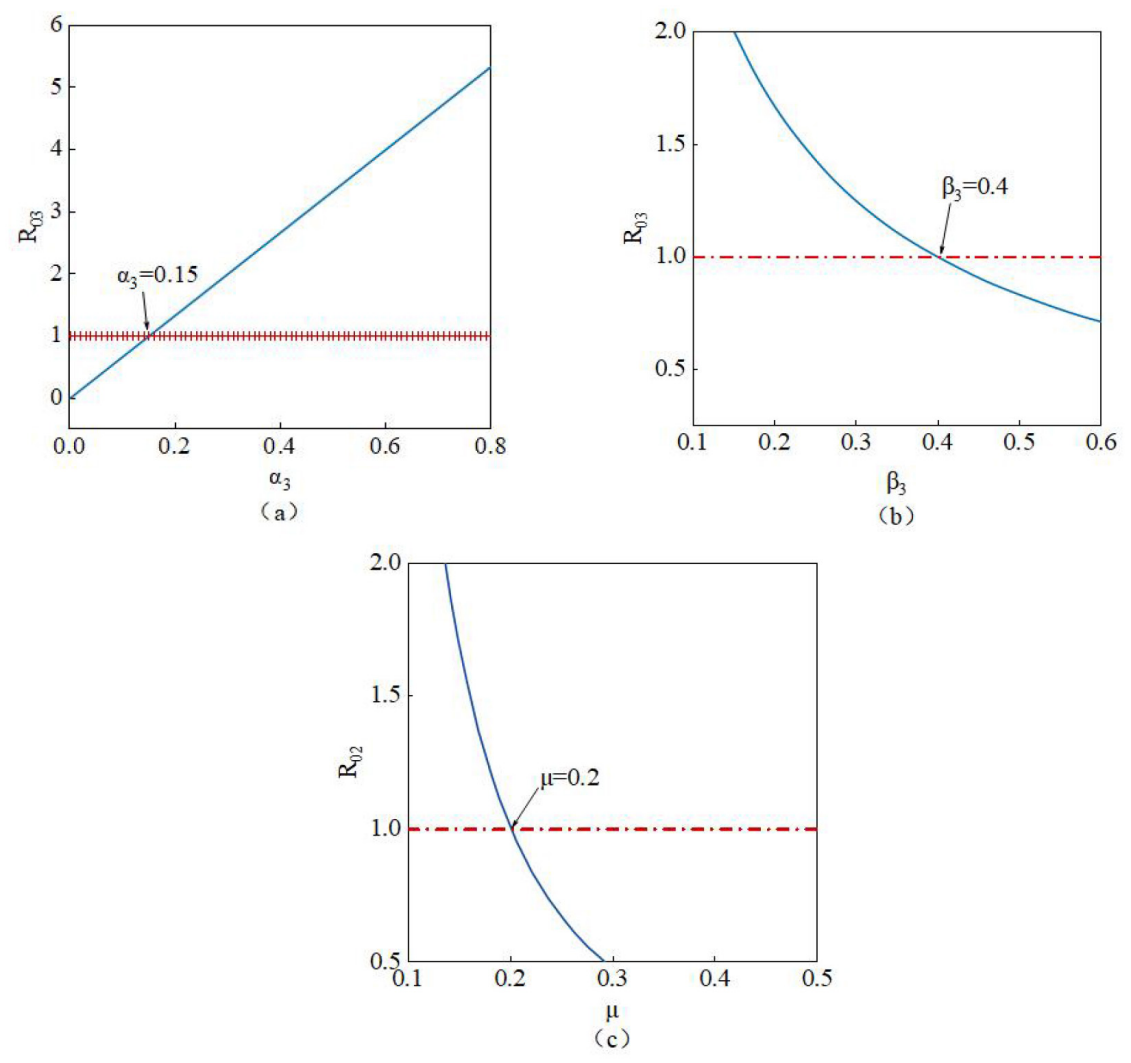
Impact of parameters **(a)** α*3*, **(b)** β*3*, and **(c)** μ on *R*03.

$R_{03}=\frac{\alpha_{3}}{\beta_{3}+\mu }$, The parameters involved are α*3*, β*3*, and μ. The simulation results from Figures 14a–c show that the value of *R*03 increases as the parameter α*3* increases. Therefore, a larger value of α*3* indicates a higher probability of susceptible nodes transforming into infected nodes. Conversely, the value of *R*03 decreases as the parameters β*3* and μ increase. Thus, higher values of β*3* and μ lead to a continuous reduction in *R*03 until it falls below 1, thereby effectively suppressing the spread of the rumor. In summary, when *R*03 < 1, the system will reach a rumor-free equilibrium state, and the spread of the rumor will be completely controlled.

### 3.4 Impact of different intervention timings on the spread of online rumors

**Figure 15:**
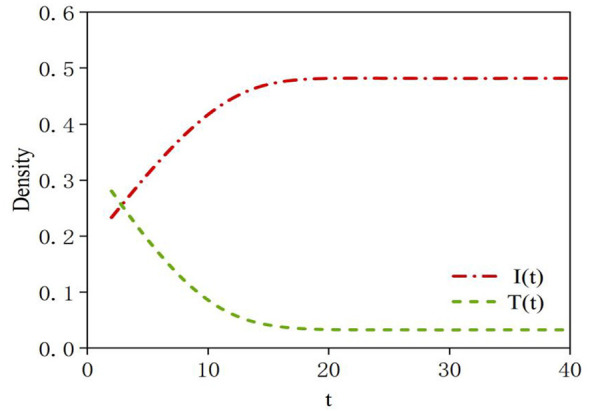
Density of infected nodes and transmitter nodes under different parameter *t* values.

**Figure 16:**
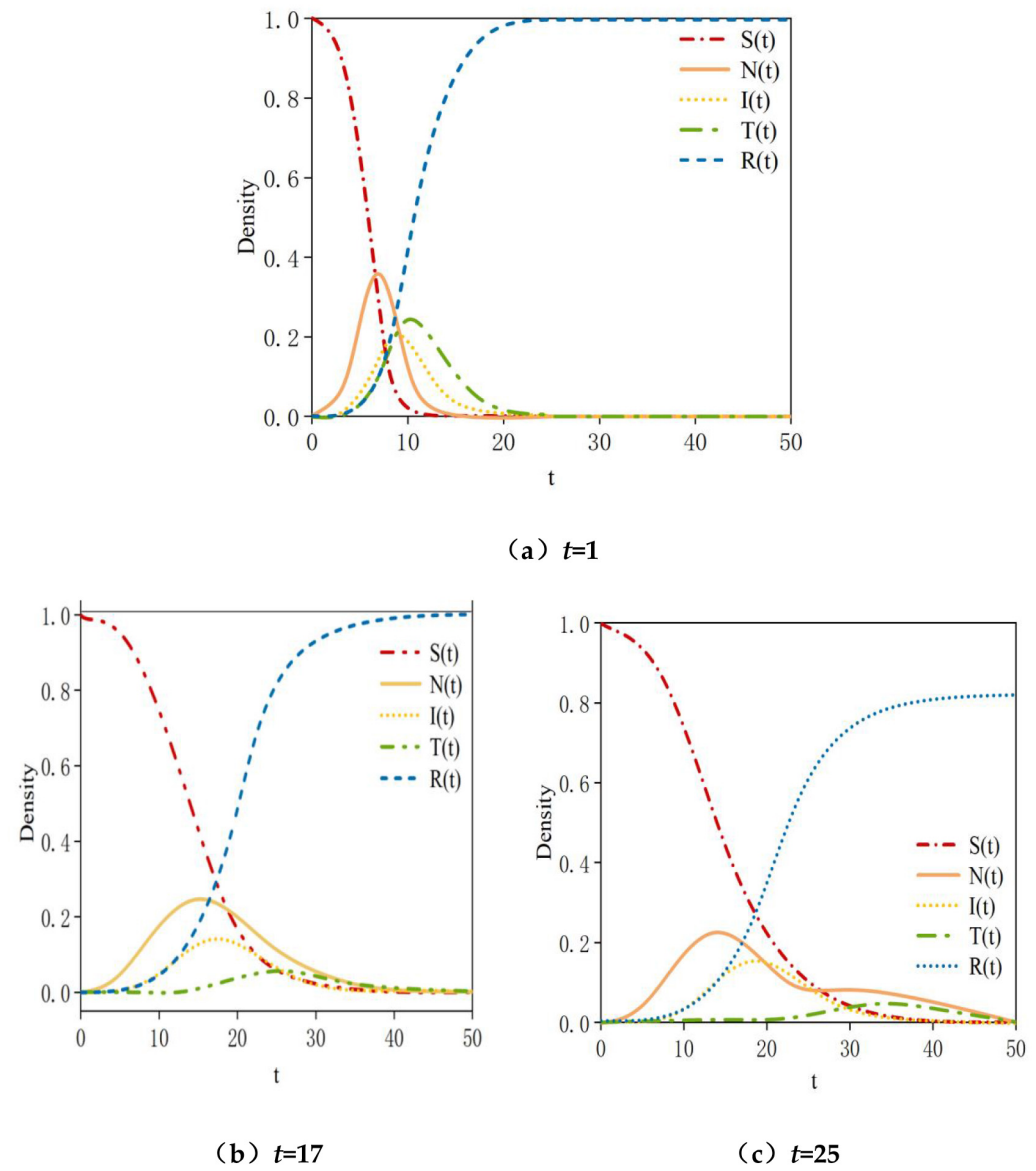
Density variation diagram of the five states in spreading authentic information. **(a)** *t* = 1; **(b)** *t* = 17; **(c)** *t* = 25.

In our study, multiple simulations were conducted by varying the value of the time parameter *t* (representing the time at which transmitter nodes enter the social network) within the range [1, 35] to investigate the impact of the timing of transmitter node entry on the dynamics of rumor propagation. As shown in Figure 15, as time *t* increases, the maximum density of infected nodes initially rises rapidly to 0.47 and then stabilizes starting from the 15th time step. Conversely, the maximum density of transmitter nodes undergoes a rapid decline, dropping to approximately 0.04, and remains relatively stable after the 15th time step. The simulation results indicate that the earlier the transmitter nodes enter the system, the greater the inhibitory effect on rumors. Notably, the optimal timing for rumor debunking is at early stage of *t* = 8, the total number of infected nodes is relatively small, while the density of users in the neglected state reaches its peak. Introducing transmitter information at this point can directly target the early source of the rumor, effectively preventing new nodes from being infected, as illustrated in Figures 5–16a. In other words, if transmission occurs after the peak, some nodes will have already transitioned from neglected to infected states, with their perceptions becoming relatively solidified and their acceptance of transmitter information reduced. Transmit efforts would then primarily affect the remaining susceptible nodes and newly generated neglected nodes, with a more limited scope of impact.

Furthermore, if there are still sufficient uninformed users when the factual information enters the system, transmit rumors before time *t* = 17, Late-stage transmit may inadvertently reignite the topic's relevance of the rumor. When some nodes are exposed to transmitter information, they may, out of curiosity or skepticism, re-search or re-discuss the original rumor, leading to a brief secondary wave of propagation. As a result, the density of neglected state nodes will experience a second peak, as shown in Figure 16b. However, if transmitting occurs at time *t* = 25, the density of neglected nodes will experience a second peak, as illustrated in Figure 16c. If the rumor undergoes changes due to distortion during its spread, it can also lead to a second peak in the density of neglected nodes.

In summary, the earlier the rumor is transmitted, the more effective the suppression of the rumor becomes. The optimal timing for transmitting should occur before the density of neglected nodes reaches its peak. If transmitting is attempted after the peak, it may not only fail to curb the spread of the online rumor but could also potentially lead to its resurgence.

## 4 Validate the model using a real-world dataset

The study utilizes a real-world data set to validate the SNITR rumor propagation model. We selected 440 rumor posts and 12,372 reposts related to topics such as “Will the Turkey earthquake trigger a strong earthquake in China?” “Are the birds gathering on Nanjing highways and birds crashing into trees in Chengdu precursors to earthquakes?”, “Does long-term mask-wearing cause lung nodules?”, “Reports of female victims”, “deaths in the Tangshan barbecue restaurant assault case”, and “Explosion at a laboratory in Tianjin”. In our research, Weibo posts labeled with status “r” are defined as rumor posts, representing the infected nodes (I) in the SNITR model. By aggregating the total number of reposting users for infected nodes (I) at 10-min intervals, we collected data from the first 30 h for parameter estimation of the model and used data from the subsequent 35 h for model validation.

To address the limitation of existing models where parameters rely on empirical settings and fail to fully integrate the reality of rumor propagation, the study employs the least squares fitting method to estimate the parameters of the SNITR model. By continuously optimizing each parameter through iterative adjustments within the range of 0.001 to 0.999, the model parameters are refined with the goal of minimizing the residual sum of squares. The final parameter estimation results are presented in Table 8.

**Table 8:** Parameter estimation.

Parameter	α_1_	α_2_	α_3_	μ	β_1_	β_2_	β_3_	ε	γ_1_	γ_2_
Estimate	0.3553	0.1168	0.4315	0.001	0.4772	0.0700	0.2351	0.0951	0.4984	0.0023

**Figure 17:**
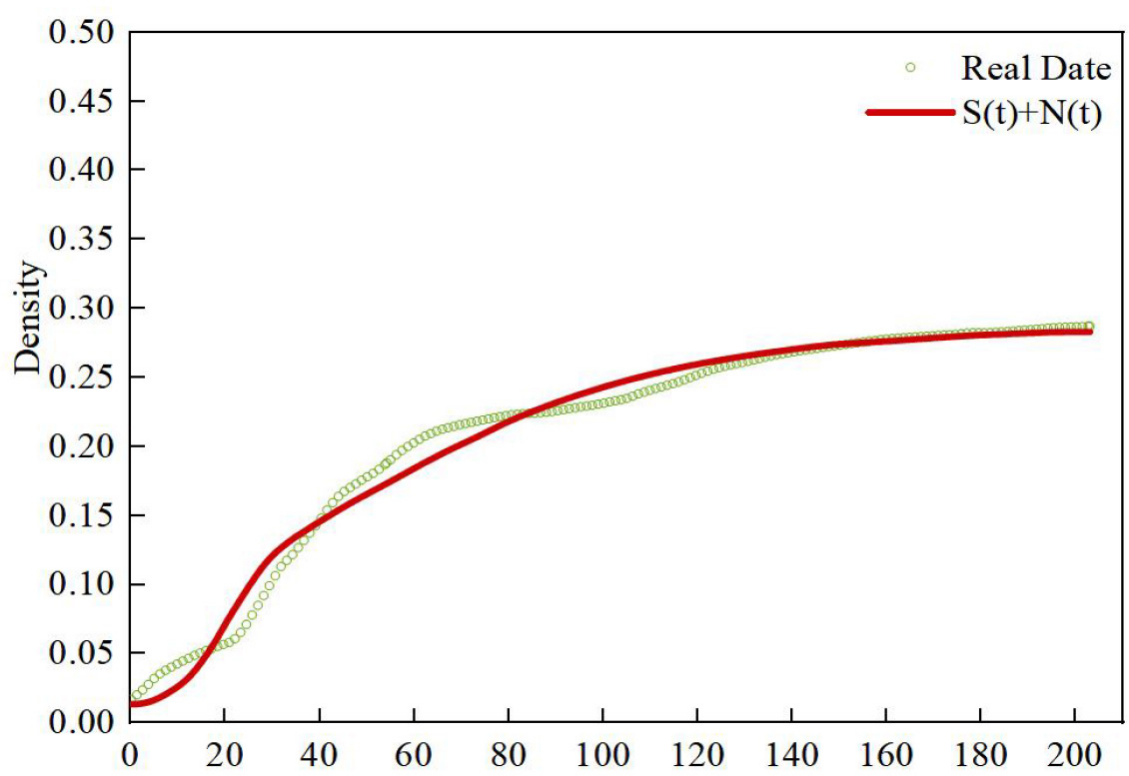
The “I + T” status curve for the first 30 h of real data.

**Figure 18:**
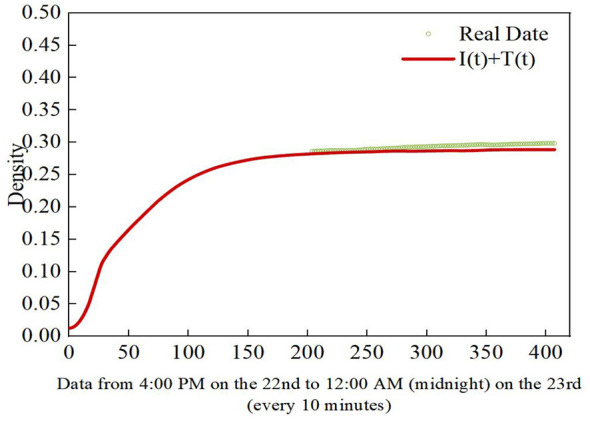
The “I + T” status curve for the last 35 h of real data.

Figures 17, 18 show the comparison between the predicted “I + T” density distribution of the SNITR model under optimal fitting conditions and the actual data at different periods. In the figures, the scatter points represent the actual data, while the red curve represents the model predictions. Figure 17 displays the “I + T” state curve based on the first 30 h of real data, where it can be observed that the model predictions align closely with the actual data. By substituting the parameter estimates from Table 8 into System (2), the density distribution of infected nodes and transmitter nodes “I + T” is simulated. Next, by comparing the scatter plot of the actual data from the last 35 h with the model predictions, as shown in Figure 18, the fitting coefficient between the actual data and the model's “I + T” density is calculated to be 0.9488. This indicates that the SNITR model can effectively predict the rumor propagation trend for the subsequent 35 h. Thus, the validity and accuracy of the model are verified.

## 5 Conclusions and suggestions

During public health events, rumors can spread rapidly and widely, exerting negative impacts on both society and individuals. Therefore, to maintain social stability and prevent potential harm to people's lives, it is crucial to curb the spread of rumors. In large-scale social networks, individuals with different characteristics hold varying attitudes toward rumors. To accurately simulate the process of rumor propagation, the study proposes a novel SNITR rumor propagation model, which conducts fine-grained modeling of user groups with different cognitive levels and dissemination tendencies. Additionally, a rumor-countering mechanism is introduced to establish a competitive coupling mechanism among different user groups.

(1) By incorporating transmitter nodes, users on a social network are classified, and a differential dynamical system is established. Neglected nodes, as users transitioning from the susceptible state to the infected state, directly determine the direction of rumor diffusion or extinction. This addresses the limitation of the “black-or-white” dichotomy in traditional rumor propagation models. Moreover, by redefining the rules of information dissemination among nodes based on the interaction between infected nodes and transmitter nodes, the newly established model better aligns with the dynamics of rumor propagation on social networks.

(2) This study first derives the equilibrium points and the basic reproduction number of the system. Based on the Routh-Hurwitz criterion and Lyapunov stability theory, a systematic analysis of the local asymptotic stability of the equilibrium points is conducted. By constructing a Lyapunov function, it is proven that the system exhibits global asymptotic stability, meaning that the system state will eventually converge to a stable point regardless of the initial conditions.

## Data Availability

The original contributions presented in the study are included in the article/supplementary material, further inquiries can be directed to the corresponding authors.
